# Diverse CRISPRs Evolving in Human Microbiomes

**DOI:** 10.1371/journal.pgen.1002441

**Published:** 2012-06-13

**Authors:** Mina Rho, Yu-Wei Wu, Haixu Tang, Thomas G. Doak, Yuzhen Ye

**Affiliations:** 1 School of Informatics and Computing, Indiana University, Bloomington, Indiana, United States of America; 2 Center for Genomics and Bioinformatics, Indiana University, Bloomington, Indiana, United States of America; 3 Department of Biology, Indiana University, Bloomington, Indiana, United States of America; University of Toronto, Canada

## Abstract

CRISPR (Clustered Regularly Interspaced Short Palindromic Repeats) loci, together with *cas* (CRISPR–associated) genes, form the CRISPR/Cas adaptive immune system, a primary defense strategy that eubacteria and archaea mobilize against foreign nucleic acids, including phages and conjugative plasmids. Short spacer sequences separated by the repeats are derived from foreign DNA and direct interference to future infections. The availability of hundreds of shotgun metagenomic datasets from the Human Microbiome Project (HMP) enables us to explore the distribution and diversity of known CRISPRs in human-associated microbial communities and to discover new CRISPRs. We propose a targeted assembly strategy to reconstruct CRISPR arrays, which whole-metagenome assemblies fail to identify. For each known CRISPR type (identified from reference genomes), we use its direct repeat consensus sequence to recruit reads from each HMP dataset and then assemble the recruited reads into CRISPR loci; the unique spacer sequences can then be extracted for analysis. We also identified novel CRISPRs or new CRISPR variants in contigs from whole-metagenome assemblies and used targeted assembly to more comprehensively identify these CRISPRs across samples. We observed that the distributions of CRISPRs (including 64 known and 86 novel ones) are largely body-site specific. We provide detailed analysis of several CRISPR loci, including novel CRISPRs. For example, known streptococcal CRISPRs were identified in most oral microbiomes, totaling ∼8,000 unique spacers: samples resampled from the same individual and oral site shared the most spacers; different oral sites from the same individual shared significantly fewer, while different individuals had almost no common spacers, indicating the impact of subtle niche differences on the evolution of CRISPR defenses. We further demonstrate potential applications of CRISPRs to the tracing of rare species and the virus exposure of individuals. This work indicates the importance of effective identification and characterization of CRISPR loci to the study of the dynamic ecology of microbiomes.

## Author Summary

Human bodies are complex ecological systems in which various microbial organisms and viruses interact with each other and with the human host. The Human Microbiome Project (HMP) has resulted in >700 datasets of shotgun metagenomic sequences, from which we can learn about the compositions and functions of human-associated microbial communities. CRISPR/Cas systems are a widespread class of adaptive immune systems in bacteria and archaea, providing acquired immunity against foreign nucleic acids: CRISPR/Cas defense pathways involve integration of viral- or plasmid-derived DNA segments into CRISPR arrays (forming spacers between repeated structural sequences), and expression of short crRNAs from these single repeat-spacer units, to generate interference to future invading foreign genomes. Powered by an effective computational approach (the targeted assembly approach for CRISPR), our analysis of CRISPR arrays in the HMP datasets provides the very first global view of bacterial immunity systems in human-associated microbial communities. The great diversity of CRISPR spacers we observed among different body sites, in different individuals, and in single individuals over time, indicates the impact of subtle niche differences on the evolution of CRISPR defenses and indicates the key role of bacteriophage (and plasmids) in shaping human microbial communities.

## Introduction

CRISPRs, together with *cas* genes (CRISPR-associated genes), provide acquired resistance against viruses and conjugative plasmids [1], [2], and are found in most archaeal (∼90%) and bacterial (∼40%) genomes [3], [4], [5]. CRISPR arrays consist of 24–47 bp direct repeats, separated by unique sequences (spacers) that are acquired from viral or plasmid genomes [6]. Even though some CRISPR arrays may contain hundreds of spacers (an extreme case is the CRISPR array in the *Haliangium ochraceum* DSM 14365 genome, which has 588 copies of its repeat), they tend to be much smaller, generally with dozens of spacers. The repeat sequences of some CRISPRs are partially palindromic, and have stable, highly conserved RNA secondary structures, while others lack detectable structures [7].

CRISPR arrays are usually adjacent to *cas* genes, which encode a large and heterogeneous family of proteins with functional domains typical of nucleases, helicases, polymerases, and polynucleotide-binding proteins. CRISPR/Cas systems commonly use repeat and spacer-derived short guide CRISPR RNAs (crRNAs) to silence foreign nucleic acids in a sequence-specific manner [8], . CRISPR/Cas defense pathways involve several steps, including integration of viral or plasmid DNA-derived spacers into the CRISPR array, expression of short crRNAs consisting of unique single repeat-spacer units, and interference with invading foreign genomes at both the DNA and RNA levels, by mechanisms that are not yet fully understood [8], [10]. The diversity of *cas* genes suggests that multiple pathways have arisen to use the basic information contained in the repeat-spacer units in diverse defense mechanisms. The CRISPR components are evolutionarily closely linked and potentially evolve simultaneously as an intact locus—sequence analysis reveals that the direct repeats in CRISPR locus and the linked *cas* genes co-evolve under analogous evolutionary pressure [11].

Previous studies have shown that CRISPR loci are very diverse and abundant in the genomes of bacteria and archaea. In addition, it has been shown that CRISPR loci with the same repeat sequence and *cas* gene set can be found in multiple bacterial species, implying horizontal gene transfer (HGT) [12]. Moreover, CRISPR loci can change their spacer content rapidly, as a result of interactions between viruses (or plasmids) and bacteria: several metagenomic studies investigating host-virus population dynamics have shown that CRISPR loci evolve in response to viral predation and that CRISPR spacer content and sequential order provide both historically and geographically insights [13], [14], [15], [16]—essentially, epidemiology.

As a reflection of the infectious dynamics of microbial communities, the study of CRISPRs is an essential compliment to the study of the human microbiome, encompassing both disease ecology and ecological immunology [17]. Infectious disease works to maintain both species diversity [18], [19] and genotypic diversity [20] within a species, as has recently been shown for marine microbiomes [21], [22]. As such, infectious agents may be at least partially responsible for the amazing species diversity and turnover found throughout the human microbiome [23]. The ability of CRISPR loci to prevent plasmid spread is medically relevant, in that the exchange of conjugative elements is perhaps the dominant mechanism by which antibiotic resistance genes (notably multi-drug resistance) move within a biome, and by which pathogens acquire resistance [24]; CRISPR activities could be expected to retard this exchange (*e.g.* [25]).

CRISPR composition in human microbial communities, the relative rate of CRISPR locus change, or how CRISPR loci vary between different body sites and between the microbiota of different individuals are less studied, as compared to other environments. A recent analysis of streptococcal CRISPRs from human saliva, in which CRISPR spacers and repeats were amplified from salivary DNA, using the conserved streptococcal CRISPR repeat sequence for priming, revealed substantial spacer sequence diversity within and between subjects over time [26], which is imagined to reflect the dynamics of phage and other infectious agents in the human mouth [2].

**Figure 1:**
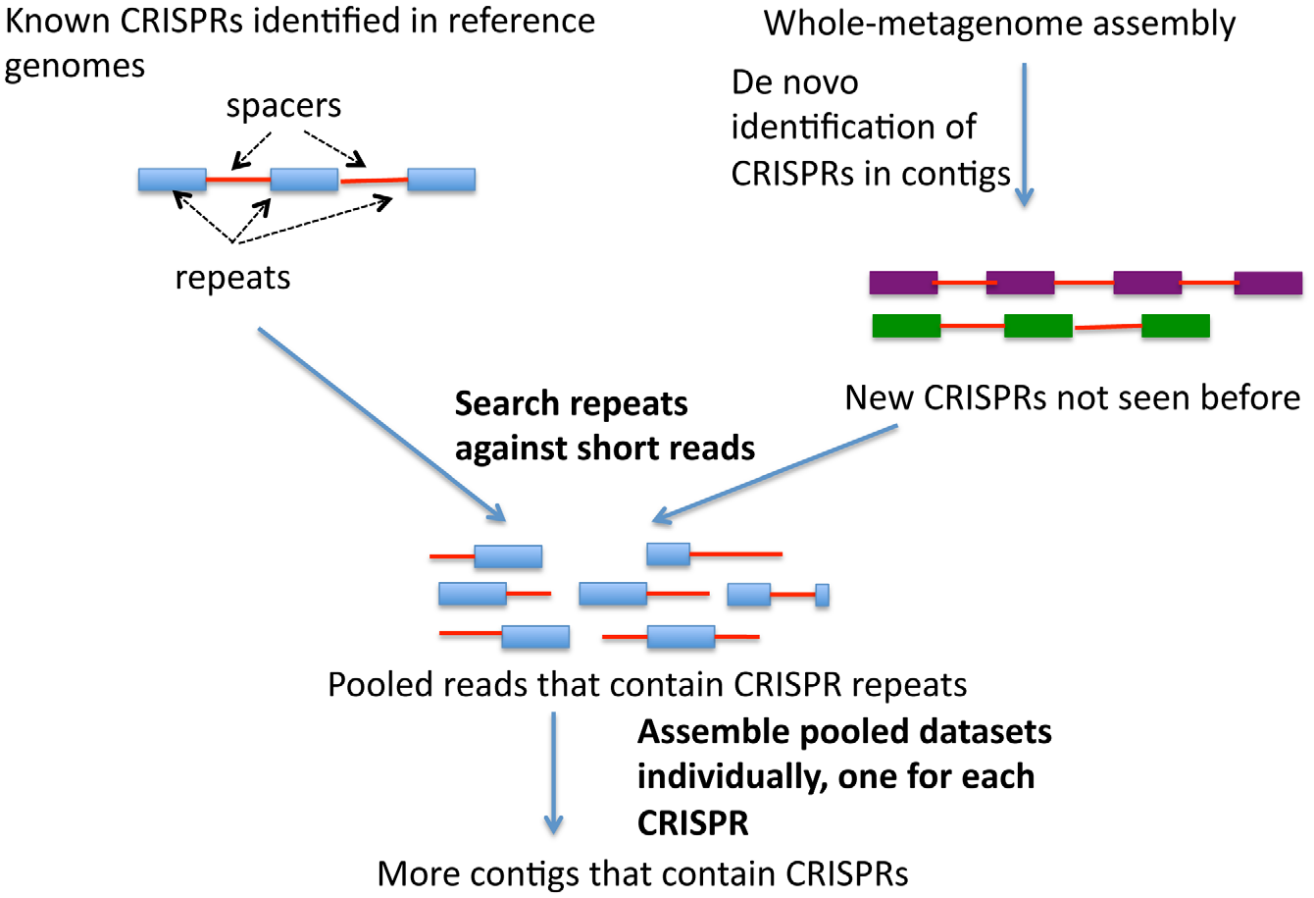
A diagram of the targeted assembly approach for CRISPR.

The availability of more than 700 shotgun metagenomic datasets from the Human Microbiome Project (HMP) enables us to explore the distribution and diversity of many more CRISPRs, and to discover new ones, across different body sites, in a systematic manner. We developed a targeted assembly strategy (see Figure 1) to better identify CRISPRs in shotgun metagenomic sequences, as whole-metagenomic assembly failed to reconstruct many CRISPRs that otherwise could be identified. All of the programs available to date [27], [28], [29], [30] are designed to find CRISPRS from assembled contigs that are sufficiently long to contain at least partial CRISPR loci; however, it is very difficult to assemble metagenome reads into contigs containing CRISPR loci, because of their repeated structures. We thus needed to collect sequencing reads associated with CRISPRs and assemble them specifically. For known CRISPRs (identified in reference genomes), we identified consensus sequences of CRISPR repeats, collected the reads containing these sequences, and assembled these reads into CRISPR contigs. We also identified CRISPRs from the whole-metagenome assemblies, and for the novel CRISPRs or new CRISPR variants (that are not seen in the reference genomes), applied the same assembly strategy to achieve a more comprehensive identification of the novel CRISPRs across the samples. This approach allows us to study the evolution of CRISPRs in human microbiomes.

## Results

We identified and selected 64 known CRISPRs—including the streptococcal CRISPR—from complete and draft bacterial genomes and 86 novel CRISPRs from the 751 HMP whole-metagenome assemblies, using metaCRT and CRISPRAlign (see Methods). For each selected CRISPR, we then applied the targeted assembly approach (for each CRISPR, first pool the reads that contain the repeat, and then assemble the pooled reads only; see Methods for a validation of the targeted assembly approach using simulated datasets) to achieve a more comprehensive characterization of the CRISPR loci in the human microbiome shotgun datasets. Below we provide detailed analysis of the targeted assembly approach, and the resulting CRISPR loci (listed in Table 1 and Tables S1 and S2).

**Table 1:** List of selected CRISPRs discussed in the paper.

ID^a^	Species (or HMP sample ID)Consensus sequence of the CRISPR repeats
**Known**	
AhydrL30	*Anaerococcus hydrogenalis* DSM 7454 (NZ_ABXA01000037)ATTTCAATACATCTAATGTTATTAATCAAC
AlactL29	*Anaerococcus lactolyticus* ATCC 51172 (NZ_ABYO01000191)AGGATCATCCCCGCTTGTGCGGGTACAAC
BcoprL32	*Bacteroides coprophilus* DSM 18228 (NZ_ACBW01000156)GTCGCACCCTGCGTGGGTGCGTGGATTGAAAC
FalocL36	*Filifactor alocis* ATCC 35896 (NZ_GG745527)TTTGAGAGTAGTGTAATTTCATATGGTAGTCAAAC
GhaemL36	*Gemella haemolysans* ATCC 10379 (EQ973306)GTTTGAGAGATATGTAAATTTTGAATTCTACAAAAC
LcrisL29	*Lactobacillus crispatus* ST1 (NC_014106)AGGATCACCTCCACATACGTGGAGAATAC
LjassL36	*Lactobacillus gasseri* JV-V03 (NZ_ACGO01000006)GTTTTAGATGGTTGTTAGATCAATAAGGTTTAGATC
LjensL36	*Lactobacillus jensenii* 115-3-CHN (NZ_GG704745)GTTTTAGAAGGTTGTTAAATCAGTAAGTTGAAAAAC
Neis_t014_L28	*Neisseria* sp. oral taxon 014 str. F0314 (NZ_GL349412)GTTACCTGCCGCACAGGCAGCTTAGAAA
Neis_t014_L36	*Neisseria* sp. oral taxon 014 str. F0314 (NZ_GL349412)GTTGTAGCTCCCTTTCTCATTTCGCAGTGCTACAAT
PacneL29	*Propionibacterium acnes* J139 (NZ_ADFS01000004)GTATTCCCCGCCTATGCGGGGGTGAGCCC
PpropL29	*Pelobacter propionicus* DSM 2379 (NC_008609)CGGTTCATCCCCGCGCATGCGGGGAACAC
SmutaL36	*Streptococcus mutans* NN2025GTTTTAGAGCTGTGTTGTTTCGAATGGTTCCAAAAC
**Novel**	
SRS012279L38	SRS012279 (dataset from a tongue dorsum sample)TATAAAAGAAGAGAATCCAGTAGAATAAGGATTGAAAC
SRS018394L37	SRS018394L37 (dataset from a supragingival plaque sample)GTATTGAAGGTCATCCATTTATAACAAGGTTTAAAAC
SRS023604L36	SRS023604 (dataset from a posterior fornix sample)GTTTGAGAGTAGTGTAATTTATGAAGGTACTAAAAC

a The IDs of the CRISPRs are assigned using the following rules: 1) If a CRISPR (*e.g.*, SmutaL36) is identified from a known complete/draft genome with species name (for SmutaL36, the genome is *Streptococcus mutans* NN2025), its ID uses five letters from the species name (*i.e.*, Smuta) followed by the length of the repeats (length of 36 is shown as L36); 2) If a CRISPR (Neis_t014_L28) is identified from a known complete/draft genome that has only general genus information (*e.g.*, *Neisseria sp*. oral taxon 014 str. F0314), then its ID is four letters from the genus name, followed by the taxon ID, and the length of the repeats; and 3) the CRISPRs identified in the HMP datasets are named as the ID of the datasets followed by the length of repeat.

### Targeted assembly improves the characterization of CRISPRs

We first asked if our targeted assembly strategy helps to identify CRISPR elements from metagenomic datasets, and found that it greatly improved detection (see comparison in Table 2). The improvements are twofold. First, the targeted assembly approach identifies known CRISPRs in more human microbiome datasets, as compared to the annotation of CRISPRs using whole-metagenome assemblies. Second, targeted assembly resulted in longer CRISPR arrays, from which we can extract many more diverse spacers for analyzing the evolution of the CRISPRs and other purposes. Here we use three examples to demonstrate the performance of the targeted assembly.

**Table 2:** Comparison of CRISPR identification using whole-metagenome assembly and targeted assembly.

		Whole-metagenome assembly		Targeted assembly		
CRISPR	Sample datasets	Spacers (max)	Spacers (total)	Short reads	Spacers (max)	Spacers (total)
SmutaL36 (386^a^ vs 38^b^)	SRS017025 (plaque)	1^c^	1^d^	1078^e^	26	76
	SRS011086 (tongue)	1	2	4018	24	78
GhaemL36 (257 versus 9)	SRS019071 (tongue)	0	0	1718	47	21
	SRS014124 (tongue)	3	3	490	21	58
SRS018394L37 (238 versus 39)	SRS049389 (tongue)	0	0	5778	25	492
	SRS049318 (plaque)	1	1	1463	38	134

a the total number of samples that have streptococcal CRISPRs identified if using targeted assembly, and

b if using whole-metagenome assembly;

c the total number of spacers found in the longest CRISPR locus found in the given dataset;

d the total number of spacers found in all contigs assembled from the given dataset;

e the total number of sequences that contain the repeats of a given CRISPR, *i.e.*, the recruited reads used for targeted assembly. See Table S1 for comparison of all the CRISPRs studied in this paper.

The first example is the streptococcal CRISPR SmutaL36 (see Table 1), a CRISPR that is conserved in streptococcal species such as *Streptococcus mutans* [26]. This CRISPR was observed only in a limited number of samples (38 out of 751 datasets) when using contigs from whole-metagenome assembly. But our targeted CRISPR assembly identifies instances of CRISPR SmutaL36 in ∼10 times more (386) datasets. Consistent with the distribution of *streptococcus* across body sites, most of the 386 datasets are from oral samples: 120 of 128 supragingival plaques (94%), 128 of 135 tongue dorsum samples (95%), and 97 of 121 buccal mucosa samples (80%) (see Table 3). CRISPR SmutaL36 was only found in a small proportion of samples from other body locations, where *streptococcus* rarely exists (*e.g.*, 4 of 148 stool samples, and none of the posterior fornix datasets). Table 2 shows the details of targeted assembly of this CRISPR in two datasets.

**Table 3:** Distribution of selected CRISPRs across body sites.

			Oral				Skin	
CRISPR	Anterior nares (94^a^)	Stool (148)	Buccal mucosa (121)	Supra-gingival plaque (128)	Tongue dorsum (135)	Posterior fornix (61)	L- (9)^c^	R- (18)^d^
SmutaL36	11^b^	4	97	120	128	0	0	1
AhydrL30	0	53	0	0	0	0	0	0
BcoprL32	0	65	0	0	0	0	0	0
FalocL36	0	63	1	18	50	0	0	0
Neis_t014_L28	0	51	15	58	15	0	0	0
Neis_t014_L36	0	0	37	66	82	0	0	0
PacneL29	1	0	0	0	0	0	4	7

a the total number of datasets;

b the total number of datasets that have CRISPRs identified;

c L-Retroauricular crease;

d R-Retroauricular crease. Note not all body sites are listed in this table.

The other two examples are GhaemL36 and SRS018394L37 (see details in Table 2). CRISPR GhaemL36 was initially identified from the genome of *Gemella haemolysans* ATCC 10379 using metaCRT. Targeted assembly further identified instances of this CRISPR in 258 oral-associated samples. The longest contig—of 3121 bases—was assembled from the SRS019071 dataset. This CRISPR array has even more repeats (48; *i.e.*, 47 spacers) than the CRISPR array in the *Gemella haemolysans* reference genome, which has 29 repeats. CRISPR SRS018394L37 (currently not yet associated with a host genome) was initially identified from the whole-metagenome assembly of SRS018394, but targeted assembly reveals the presence of this CRISPR in 238 oral-associated microbiomes. The contig that was assembled in SRS049389 is the longest one (2014 bps), containing 25 spacers.

In most cases we have tested, targeted assembly dramatically improves the identification of CRISPRs in the HMP datasets: for 142 CRISPRs (out of 150), targeted assembly resulted in CRISPR identification in more HMP samples as compared to using whole-metagenome assemblies, and for 36 CRISPRs, targeted assembly identified instances of the corresponding CRISPR in at least 10 times more datasets (see Table S1). It suggests that specifically designed assembly approaches, such as the targeted assembly approach for CRISPR assembly presented here, are important for the characterization of functionally important repetitive elements that otherwise may be poorly assembled in a whole-metagenome assembly (which tends to be confused by repeats), and such a comprehensive identification is important for deriving an unbiased distribution of these functional elements across different body sites among individuals.

### Novel CRISPRs are found in human microbiome samples

In order to identify novel CRISPR loci, with which to seed further targeted assemblies, we set out to find loci based simply on the structural patterns of CRISPR loci, using the program metaCRT, which we modified from CRT (see Methods). As a result, we found and selected 86 different types of novel CRISPR repeats in metagenomic samples, which could not be found in reference genomes, for further targeted assembly (see Methods). Table 1 lists selected examples of novel CRISPRs that we identified in HMP datasets (see Table 1 for naming conventions). A full list of CRISPRs (including the number of CRISPR contigs assembled in each metagenomic dataset) is available as Table S1. In this section, we highlight two examples of novel CRISPRs.

**Figure 2:**
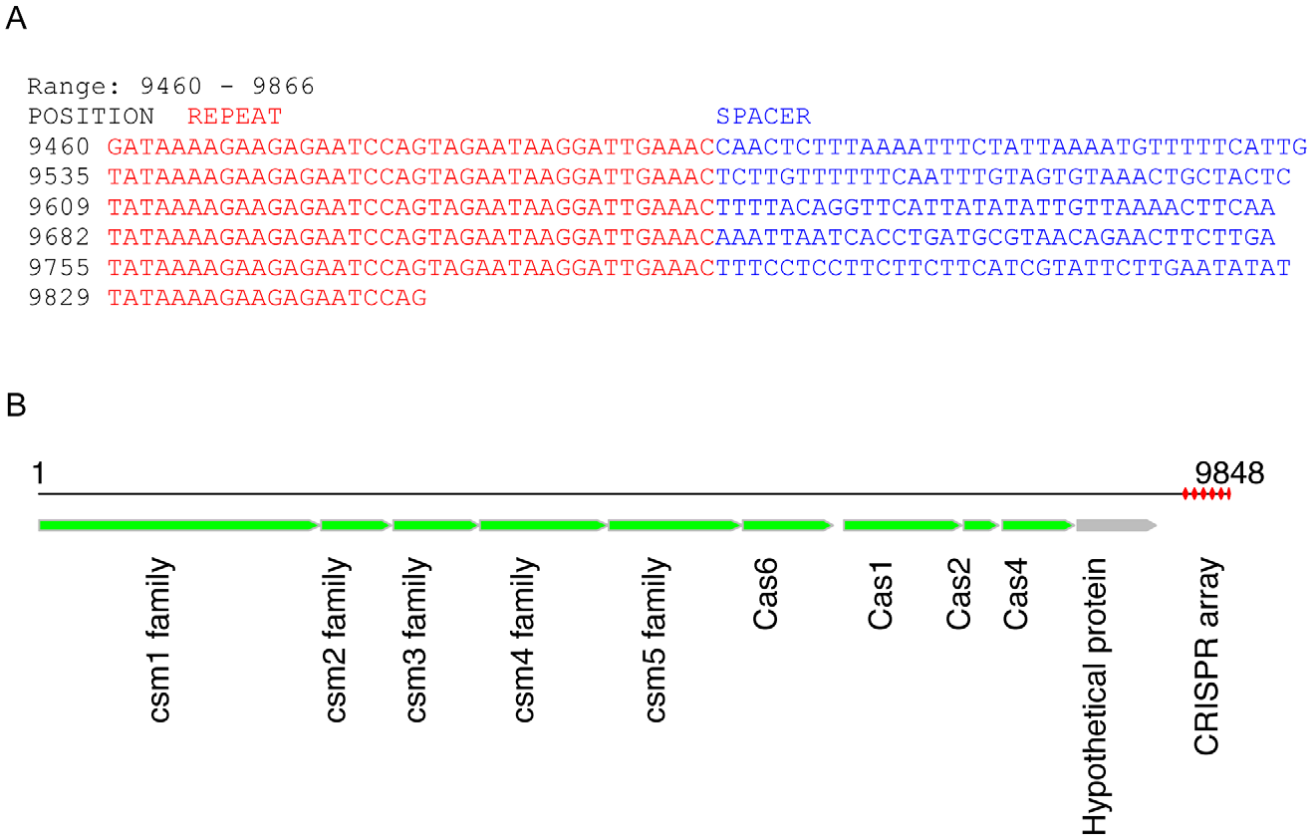
A potentially novel CRISPR array identified in a contig (9848 bases) from sample SRS012279. (A) This CRISPR array has 6 copies of the repeat (repeat sequences shown in red font and spacer shown in blue). (B) shows our annotation of this contig, in which the CRISPR array is highlighted in red. We first predicted ORFs in this contig using FragGeneScan [31], and then blasted predicted proteins against the nr protein database to retrieve annotations; for example, the predicted Cas1 is similar to the Cas1 protein identified in *Leptotrichia buccalis* C-1013-b (accession ID: YP_003163976), with 60% sequence identify and 80% sequence similarity.

CRISPR SRS012279L38 was identified from a whole-metagenome assembly contig of dataset SRS012279 (derived from a tongue dorsum sample; see Figure 2A). The identified CRISPR contig has 6 copies of a 38-bp repeat (the last copy is incomplete; see Table 1 for the consensus sequence of the repeats). *De novo* gene prediction by FragGeneScan [31] reveals 10 protein-coding genes in this contig, among which 9 share similarities with *cas* genes from other genomes, including *Leptotrichia buccalis* DSM 1135 (NC_013192, an anaerobic, gram-negative species, which is a constituent of normal oral flora [32]) and *Fusobacterium mortiferum* ATCC 9817, by BLASTP search using the predicted protein sequences as queries (see Figure 2B). (By contrast, BLASTX search of this contig against nr database only achieved annotations for 7 genes). In addition, similarity searches revealed a single identical copy of this repeat in the genome of *Leptotrichia buccalis* DSM 1135 (from 1166729 to 1166764; *de novo* CRISPR prediction shows that this genome has several CRISPR arrays, including an array that has 84 copies of a 29-bp repeat, but none of the CRISPRs have the same repeat sequence as SRS012279L38). These two lines of evidence (similar *cas* genes, and an identical region in the genome) suggest that the SRS012279L38 CRISPR we found in the human microbiomes could have evolved from *Leptotrichia buccalis* or a related species.

Targeted assembly of this novel CRISPR (SRS012279L38) in HMP datasets resulted in 278 contigs from 97 datasets, confirming the presence of this CRISPR in human microbiomes. In particular, the CRISPR fragments (407 bps) identified from the whole-metagenome assembly of SRS012279 were assembled into a longer CRISPR contig (890 bps) by targeted assembly. A total of 14 unique but related repeat sequences were identified from 278 CRISPR contigs, and two of them (which differ at 3 positions) are dominant, constituting 71% of the repeats in the CRISPR contigs. Notably, all the repeats could be clustered into a single consensus sequence with an identity threshold of 88%. By contrast, the spacer sequences are very diverse across different samples. For example, we obtained a total of 352 unique spacer sequences, which were clustered into 345 consensus sequences with an identity threshold of 90%. Among 352 unique spacers, 114 spacer sequences were shared by multiple samples—a single spacer was shared by at most eight samples.

The second example is CRISPR SRS023604L36, initially identified in a whole-metagenome assembly contig of dataset SRS023604 (derived from posterior fornix), which has 5 copies of a 36 bp repeat (see consensus sequence of the CRISPR repeat in Table 1). Targeted assembly of this CRISPR across all HMP metagenomic datasets revealed further instances of this CRISPR in several other datasets, including two from stool, and two from posterior fornix. Moreover, the CRISPR contig was assembled into a longer contig of 778 bps containing 12 copies of the CRISPR repeat. BLAST search of the CRISPR repeat against the nr database did not reveal any significant hits.

### Expanding the CRISPR space by human microbiomes

**Figure 3:**
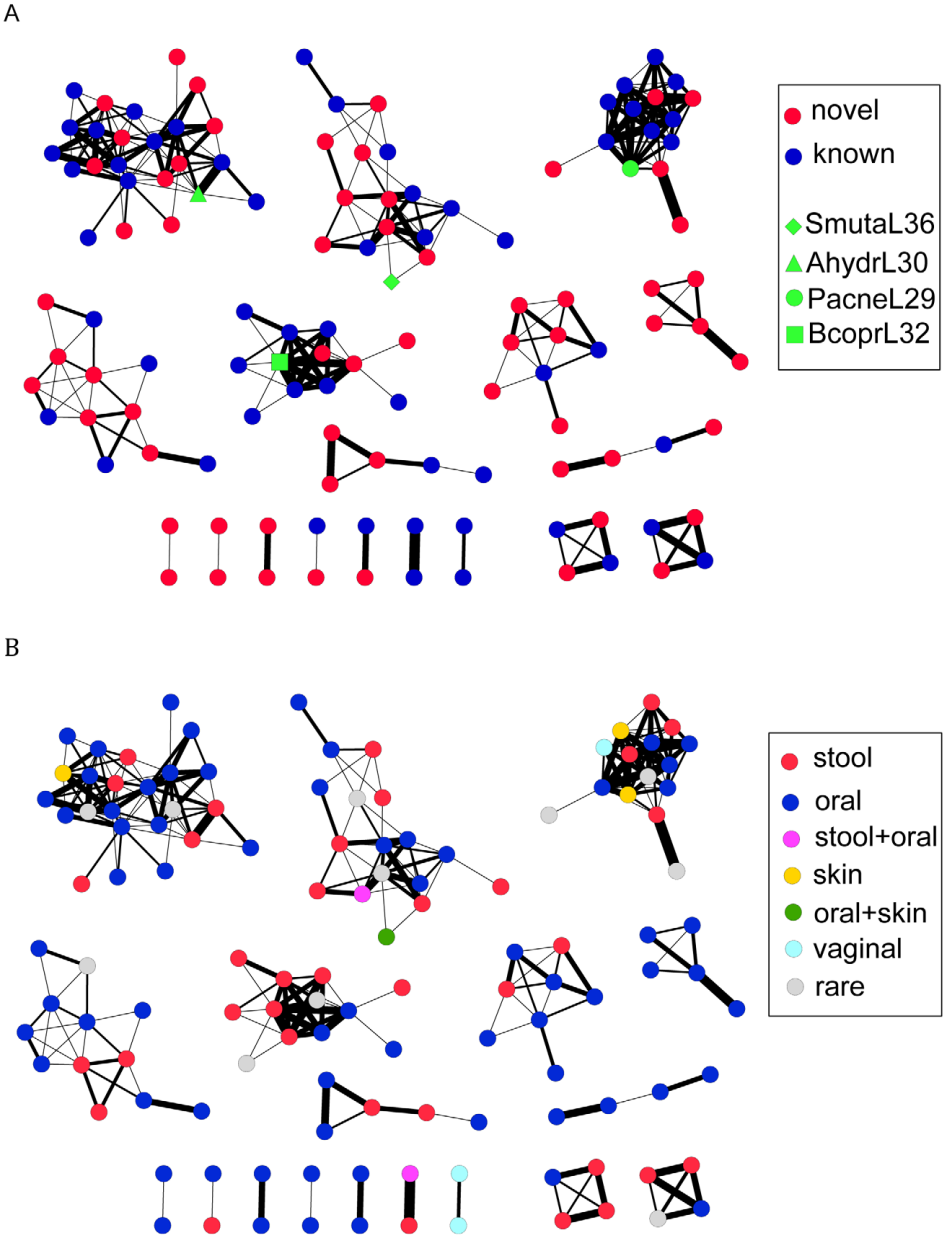
Visualizations of the CRISPR network of 150 CRISPRs, each represented as a node. There is an edge between two nodes, if the edit distance between the consensus sequences of the repeats of the corresponding CRISPRs is <10, with edges of small edit distances (*i.e.*, the two CRISPRs share more similar repeats) shown in thick lines and edges of larger edit distances in thin lines. In (A), the known CRISPRs are shown as blue nodes (except for several CRISPRs highlighted in green), and the novel CRISPRs identified in the HMP datasets are shown as red nodes. In (B), the nodes are colored based on body site, in which the CRISPRs are most frequently found. CRISPRs are assigned as rare if they were found in <5 samples; otherwise, they are assigned to particular body site(s) if they are found in more than 10 percent of the samples for that particular body site (e.g., stool+skin). The figures were prepared using Cytoscape [43].

To investigate how much the CRISPRs identified in the HMP datasets can expand the CRISPR space (sequence space of the CRISPR repeats), we built a network of CRISPRs, based on the sequence similarity between CRISPR repeats. An edge in the network between two CRISPR repeats, each represented by a node, indicates that the two repeats can be transformed from one to another by at most 10 operations (including mutations, insertions, and deletions). Since it is difficult to determine the direction of CRISPR repeats [7] (especially for the CRISPR arrays that have incomplete structures), given two repeats, we calculated two edit distances—one is the distance between the two repeats, and the other one is between one repeat and the reverse complement of the other—and used the smaller value as the edit distance between the two repeats. The global network (Figure 3A; see Figure S1 with node labels) shows that most of the novel CRISPRs identified in the human microbiomes are remotely related to ones identified in complete (or draft) genomes. Still, there are small clusters that contain only novel HMP CRISPRs, indicating that these CRISPRs are substantially different from ones identified in the reference genomes. In Figure 3B, we have colored nodes by body site: while specific CRISPR repeats can be highly specific to body site (see below), the larger families of repeats shown in Figure 3B do not appear to cluster based on body site.

We further studied the sequence patterns of the repeats for each group and our results show 1) distinct patterns among the groups, and 2) high conservation around the stem and start/end positions in CRSIPR repeats of each group (see sequence logos—for the large groups—in Figure S2). The consensuses revealed by the logos show consistencies with the results in a previous study, which used a similar approach, based on alignments of CRISPR repeats, for classification of CRISPR repeats [7].

### CRISPRs have diverse distributions across human body sites and individuals

**Figure 4:**
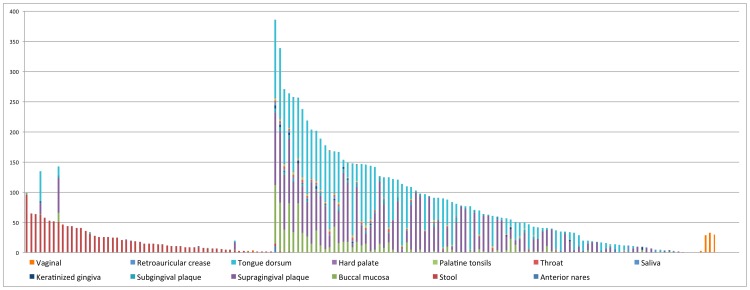
Distribution of CRISPRs across body sites. In this figure, the x-axis represents 150 CRISPRs and the y-axis represents the total number of samples in which instances of each CRISPR are found. Note that there are roughly one third as many stool samples as oral samples, probably explaining the apparently smaller number of CRISPRs in the gut microbiome. See Table S3 for details of the distribution of CRISPRs across body sites.

Overall, the distributions of CRISPRs are largely body-site specific (see Figure 4 and Figure S3; the name of CRISPR and the number of samples in which the CRISPR was found are listed in Table S3). For example, CRISPRs AhydrL30 ad BcoprL32 are only found in stool samples (see Table 3). Exceptions include two CRISPRs that were found from both a significant number of gut- and oral-associated samples: Neis_t014_L28 were found in 51 gut samples and 92 oral-associated samples; FalocL36 identified from *Filifactor alocis* ATCC 35896 were found in 63 gut samples and 72 oral-associated samples, including 50 tongue dorsum samples (see Table 3).

The first 50 CRISPRs shown in Figure 4 are mainly found in stool samples. AshahL36, which was initially identified from *Alistipes shahii* WAL 8301, was found in more than half of gut-related samples (96 out of 147 samples). On the other hand, 99 CRISPRs are mainly found in oral samples, in particular, tongue dorsum, supragingival plaque, and buccal mucosa. We found 5 CRISPRs that exist in more than half of the oral-associated samples (out of 417): SmutaL36, KoralL32 from *Kingella oralis* ATCC 51147, Veil_sp3_1_44_L36 and Veil_sp3_1_44_L35 from *Veillonella sp.* 3_1_44, and SoralL35 from *Streptococcus oralis* ATTC 35037. 4 CRISPRs are mostly found in vaginal samples (AlactL29, LjensL36, LjassL36, and LcrisL29). 1 CRISPR is skin-specific (PacneL29), found mainly in skin samples. Below we discuss the body-site distributions of a few examples.

Neis_t014_L28 and Neis_t014_L36 are inferred from a single genome, *Neisseria sp.* oral taxon 014 str. F0314, but these two CRISPRs show distinct absence/presence profiles across body sites (see Table 3). For stool samples, there exists only CRISPR Neis_t014_L28 in 51 datasets, but not Neis_t014_L36. And Neis_t014_L36 is relatively more prevalent in oral-associated samples as compared to Neis_t014_L28. The different body site distributions can be explained by the fact that these two CRISPRs are found in different sets of genomes (although both can exist in a common genome, *Neisseria sp.* oral taxon 014 str. F0314). Neis_t014_L36 has been identified in multiple *Neisseria* genomes, including *Neisseria meningitidis* ATCC 13091, *Neisseria meningitidis* 8013 (so Neis_t014_L36 belongs to the Nmeni subtype among the 8 subtypes defined by Haft et al [33]), *Neisseria flavescens* SK114, and *Actinobacillus minor* NM305. Neis_t014_L28, however, was only found in *Neisseria sp.* oral taxon 014 str. F0314. On the other hand, even though we could not find any CRISPRs containing the exactly same repeat as Neis_t014_L28 in the complete/draft genomes other than *Neisseria sp.* oral taxon 014 str. F0314, many CRISPRs, when a few mismatches are allowed, were found in diverse genomes (for example, *Crenothrix polyspora*, *Legionella pneumophila* 2300/99 Alcoy, and *Thioalkalivibrio sp.* K90mix) from environmental samples.

Four CRISPRs (AlactL29, LjensL36, LjassL36, and LcrisL29) exist mostly in vaginal samples. AlactL29, initially identified from the *Anaerococcus lactolyticus* genome, was found only in 3 vaginal samples. Notably, LjensL36 was found in 28 vaginal samples (which comprise 43% of vaginal samples collected) and 1 skin sample. This observation is consistent with a previous study showing that *Lactobacillus* constitutes over 70% of all bacteria sampled from vaginas of healthy, fertile women, and *Lactobacillus jensenii* is one of the major genomes [34]. Interestingly, we could find evidence of adaptation in the LjensL36 spacer against *Lactobacillus* phage Lv-1 (NC_011801) (see below). LjassL36 was found in 33 vaginal samples by targeted assembly. We confirmed that it is in different *Lactobacillus* genomes, such as *Lactobacillus gasseri* and *Lactobacillus crispatus*, by BLAST search. CRISPR LcrisL29, which was identified in the *Lactobacillus crispatus* genome, was found in 31 vaginal samples, and we found the same repeat sequence in the *Lactobacillus helveticus* genome.

PacneL29 was the only skin-specific CRISPR we found in the HMP datasets. Interestingly, instances of PacneL29 are found in *Propionibacterium acnes* HL110PA4 and *Propionibacterium acnes* J139, but not other *P. acnes* isolates (including KPA171202, SK137, J165, and SK187). This indicates a potential application of CRISPRs in the characterization of specific stains for a species in human microbiomes.

### CRISPRs have very diverse spacers

The HMP project enables us to explore the diversity of streptococcal CRISPRs (and others) at a much broader scale (with 751 samples from 104 healthy individuals). The CRISPRs that we identified in human microbiomes exhibited substantial sequence diversity in their spacers among subjects. Targeted assembly of the streptococcal CRISPRs (SmutaL36) in HMP datasets resulted in a total of 15,662 spacers identified from 386 samples, among which 7,815 were unique spacers (clustering of the spacers at 80% identify resulted in a non-redundant collection of 7,436 sequences). See Figure S4 for the sharing of the spacers in streptococcal CRISPRs among all individuals, which shows several large clusters of spacers that are shared by multiple individuals (for clarity, we only keep spacers that were shared by more than eight samples in this figure). In particular, the most common spacer is shared by 25 individuals (in 32 samples).

**Figure 5:**
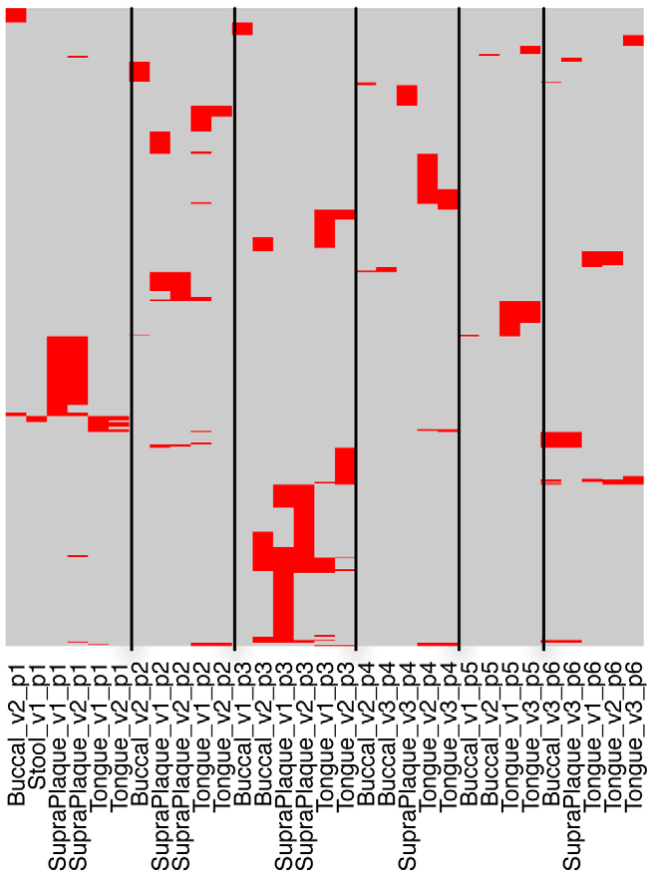
Sharing of streptococcal CRISPR spacers among samples from 6 individuals. In this map, the rows are the 761 spacers (clustered at 98% identify) identified in one or more of these 6 individuals, and the columns are samples (*e.g.*, Stool_v1_p1 indicates a sample from stool of individual 1, in visit 1; Tongue_v2_p1 indicates dataset from tongue, individual 1, in visit 2). Buccal stands for buccal mucosa, and SupraPlaque stands for supragingival plaque. The red lines indicate the presence of spacers in each of the samples. Multiple lines in the same row represent a spacer that is shared by multiple samples.

More importantly, we could check the sharing of CRISPR spacers across different body sites and sub-body sites (*e.g.*, multiple oral sites) using HMP datasets (Pride *et al.* examined streptococcal CRISPRs in saliva samples from 4 individuals [26]). Figure 5 shows the spacer sharing among 6 selected individuals, each of whom has multiple samples with identified streptococcal CRISPRs from multiple body sites (see Figure S5 for the spacer sharing with spacers clustered at 80% sequence identify). By examining the distribution of the spacers across samples, we observed that samples re-sampled from the same individual and oral site shared the most spacers, different oral sites from the same individual shared significantly fewer, while different individuals had almost no common spacers, indicating the impact of subtle niche differences and histories on the evolution of CRISPRs. Our observation is largely consistent with the conclusion from Pride *et al.* [26]. But our study showed that different samples from the same oral site of the same person, even samples collected many months apart, could still share a significant number of spacers (*e.g.*, the supragingival plaque samples from individual 1 in visit 1 and visit 2, with 238 days between the two visits, and the tongue dorsum samples from individual 5 in visit 1 and visit 3, with 336 days between the two visits; as shown in Figure 5). Our study also showed that although the different oral sites of the same individual share similar spacers, this sharing (*e.g.*, between the supragingival plaque sample and the buccal mucosa sample for individual 1) is minimal, as compared to the spacer sharing between samples collected in different visits but from the same oral site (*e.g.*, between the supragingival plaque samples from visit 1 and visit 2 for individual 1). Finally, our study shows that the spacer turnover varies among individuals—for the 6 selected individuals, individual 3 shows significantly higher turnover of the spacers between visits, as compared to other individuals.

We also checked the spacer diversity for the CRISPR KoralL32, since it and its variants are one of the most abundant CRISPRs. This CRISPR was assembled from 339 samples: 327 from oral sites and 2 from gut. The targeted assembly of KoralL32 found 7282 unique spacers, among which the most commonly shared spacer is shared by 35 individuals (in 58 samples). Figure S6 shows the sharing of the spacers among the individuals for this CRISPR, which shows similar spacer-sharing patterns as those found in the streptococcal CRISPRs.

The similarity between spacers from the same individual suggests that we may still be able to trace the evolution of CRISPRs, especially in the same body site of the same individual, even though the CRISPR loci tend to have extremely high turnover of their spacers.

### CRISPR spacer sequences can be used to trace the viral exposure of microbial communities

**Figure 6:**
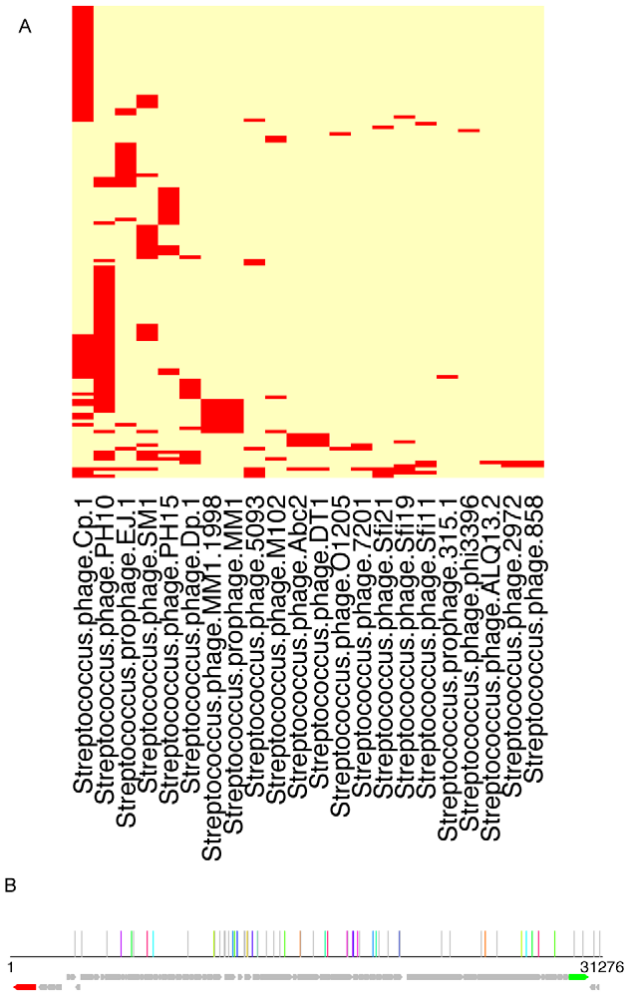
Traces of viral sequences in the streptococcal CRISPRs in human microbiomes. (A) A two-way clustering of viral genomes and the HMP datasets based on the presence patterns of viral sequences in the CRISPR loci identified in the HMP datasets: the columns are the viral genomes, and the rows are HMP datasets. It shows that the genome of *Streptococcus phage* PH10 (NC_012756) has the most regions that are similar to the spacers in streptococcal CRISPRs. This figure was prepared using the heatmap function in R, with the default clustering method (hclust) and distance measure (Euclidean). (B) Mapping of the spacers onto the 31,276 base genome of *Streptococcus phage* PH10; in this figure, each vertical line shows a potential proto-spacer, a region in the virus genome that is similar to a spacer found in HMP datasets; lines of the same color show sets of proto-spacers identified from the same HMP dataset (other individual proto-spacers are shown in gray lines); the ORFs are shown in arrows (the red arrow is an integrase and the green arrow is annotated as endolysin).

As a consequence of CRISPR adaptation, the spacer contents in CRISPR arrays reflect diverse phages and plasmids that have passed through the host genome [1], [35], [36], [37]. However, previous studies have shown that only 2% of the spacer sequences have matches in GenBank, which is probably due to the fact that bacteriophage and plasmids are still poorly represented in databases [13], [14]. Similarity searches of identified spacers against viral genomes enable identification of the viral sources of the spacers (*i.e.*, proto-spacers) captured in each CRISPR locus. For example, similarity searches of the 7,815 unique spacers in the streptococcal CRISPR against viral genomes revealed similarities between streptococcal spacers and 22 viral genomes (species names and accession IDs are listed in Table S4), and the two most prevalent viruses are *Streptococcus* phage PH10 (NC_012756) and *Streptococcus* phage Cp-1 (NC_001825) (see Figure 6A). Figure 6B suggests that the potential proto-spacers are rather evenly distributed along the phage genomes (except for a few regions, including a region that encodes for an integrase, which is highlighted in red in Figure 6B). Although the positional distribution of the proto-spacers is close to random, the sequences adjoining the proto-spacers for streptococcal CRISPR we identified in the virus genomes showed conserved short sequence motifs (GG) (see Figure S7 for the sequence logo), which is also the most common proto-spacer adjacent motif (PAM) shared by several CRISPR groups, as reported in [38].

Another example is CRISPR PacneL29, which is mainly found in skin-associated microbiomes. BLAST search of the identified spacers against the virus genome dataset revealed similarity between the spacers and several regions in *Propionibacterium* phage PA6 (NC_009541). We also found evidence of adaptation in LjensL36 against *Lactobacillus* phage Lv-1 (NC_011801): BLAST search shows significant matches to a total of 38 regions in the phage genome. Overall we found 23 CRISPRs that have spacers with high sequence similarities (≥90% over 30 bps) with virus genomes collected from the NCBI ftp site (Table S5).

We also searched the spacers against plasmid sequences (collected in the IMG database). For example, matches were found between the detected streptococcal CRISPR spacers and more than 10 plasmid sequences (including *Streptococcus thermophilus* plasmid pER35, pER36, pSMQ308, and pSMQ173b; *Bacillus subtilis* plasmid pTA1040; and *Streptococcus pneumoniae* plasmids pSMB1, pDP1 and pSpnP1). See Table S6 for a summary of the plasmids that share high homology with the CRISPR spacers.

The CRISPER spacers can also be used to identify viral contigs in metagenome assemblies that contain proto-spacers. As an example, similarity searches of identified streptococcal CRISPR spacers against the HMP assemblies revealed 37 potential viral contigs (of lengths from 2,134 to 56,413 bp): these contigs show high homology (>80% sequence similarity) with known viral genomes. The largest contig (of 56,413 bps) is similar to the genome of *Streptococcus* phage Dp-1 (NC_015274), with 88% sequence identify, and covers almost the entire viral genome (of 59,241 bps). A future paper will fully explore this approach.

### Conserved CRISPR repeat sequences can be used to reveal rare species in human microbiome

Because of the large number of repeats that many CRISPR loci contain, CRISPR repeats of rare species with low sequence coverage in a community can still be found. It was reported that repeat-based classification [7] corresponds to a *cas* gene-based classification of CRISPRs [33], which revealed several subtypes of CRISPRs largely constrained within groups of evolutionarily related species (*e.g.*, the Ecoli subtype). As such, we may use the presence of the repeats of a particular CRISPR as a first indication of the presence of related genome(s) in a microbiome, even though CRISPR locus has been found transferred horizontally as a complete package among genomes [11].

We use CRISPR PpropL29 as an example to demonstrate this potential application, as PpropL29 was identified in only a small proportion of the HMP samples (11 datasets): including 7 supragingival plaque samples (out of 125) and 4 tongue dorsum samples (out of 138). All the PpropL29-related repeats identified in these samples can be clustered into 7 unique sequences. In order to find the most likely reference genomes for these 7 unique repeat sequences, we blasted these repeat sequences against the human microbiome reference genomes and found 100% identity matches in the *Lautropia mirabills* genome. To investigate the overall coverage of this genome by the reads (not only the CRISPR regions), we mapped the entire collection of reads from four samples: SRS019980 and SRS021477 (both are from supragingival plaque, and have an 100% identity match with the CRISPR repeat in the *Lautropia mirabills* genome); SRS019974 (from tongue dorsum, with a slightly different CRISPR repeat sequence with 3 differences); and SRS019906 (which does not contain any CRISPR repeats similar to PpropL29, used as a control). The mapping results show the reads from two samples SRS019980 and SRS021477 each cover ∼80% of the *Lautropia mirabills* genome, which is very significant evidence that these two microbiomes include *Lautropia mirabills*. But the other two samples have only a limited number of reads mapped to the genome (*e.g.*, only 3089, reads in SRS019906 were mapped into *Lautropia mirabills*). This contrast suggests that identification of CRISPRs by targeted assembly could provide significant evidence for the existence of certain rare genomes.

## Discussion

We have applied a targeted assembly approach to CRISPR identification, to characterize CRISPRs across body sites in different individuals. Our studies show that a directed approach—such as our targeted assembly approach—is important for a comprehensive (thus less biased) estimation of the distribution of CRISPRs across body sites and individuals, and their dynamics. Note that in this study, we only focused on CRISPRs identified in eubacterial genomes, since archaea are rare in human microbiomes (we looked for, but did not find, archaeal CRISPRs in the HMP assemblies). Also for the sake of simplicity, we derived a non-redundant list of CRISPRs based on the similarity of the CRISPR repeats (see Methods), and detailed targeted assembly was only applied to these CRISPRs.

Although many CRISPR arrays may be missed by whole-metagenome assembly, we show that whole-metagenome assemblies are useful for identifying novel CRISPRs (as *de novo* prediction of CRISPRs relies on sequence features of CRISPRs that do not exist in short reads). Once seeding CRISPRs are identified from whole-metagenome assemblies, we can go back to the original short read datasets, and pursue a comprehensive characterization of the CRISPRs, using the targeted assembly approach. Also, we did not fully utilize the presence of *cas* genes for identification of novel CRISPRs in our study, since in many cases we can identify arrays of repeats, but not their associated *cas* genes. A future direction is to combine targeted assembly of CRISPRs and whole-metagenome assembly, aiming to achieve even better assembly of the CRISPR loci with more complete structures, including *cas* genes and the arrays of repeats and spacers. Such an improvement is necessary to achieve a more comprehensive characterization of especially the novel CRISPRs discovered in metagenomes, and the temporal order of spacer addition to arrays.

The immediate utility of this study is to provide more complete inventories of CRISPR loci in human microbiomes and their distributions in different human body sites, and the spacer content of these loci. The identification of CRISPR spacers opens up several potential applications, including tracing the viral exposure of the hosts, studying the sequence patterns of the regions adjoining the spacer precursors in viral genomes, and discovering viral contigs in metagenome assemblies. It has been shown that short sequence motifs found in the regions adjacent to the spacer precursors in the viral genomes determine the targets of the CRISPR defense system [38], and we were able to analyze the sequence patterns of regions adjacent to spacer precursors for several CRISPRs with the most spacers identified in the HMP datasets (including SmutaL36, LjensL36, and KoralL32; see sequence logos in Figure S7). When more metagenomic datasets become available, we will extend the analysis to more CRISPRs, which may provide insights into the mechanism of the CRISPR defense system (including the turnover patterns of the CRISPR spacers, and the target recognition of the CRISPR defense systems). Our preliminary exploration of viral contigs—by searching CRISPR spacers against whole-metagenome assemblies—suggests that we can identify new virus genomes in metagenome assemblies; further computational and experimental analysis will be needed to confirm these contigs.

We look forward to being able to utilize CRISPR spacer sequences to understand human and human microbiome biology better, utilizing the metadata associated with the HMP datasets. This awaits a more complete sampling of individuals over time, and of known relationships; and a far better characterization of bacteriophage and other selfish genetic elements in the human biome (our inventory of spacers is a standard against which phage and plasmid collections can be judged).

## Methods

### *De novo* identification of CRISPRs

CRT [28] is a tool for fast, *de novo* identification of CRISPRs in long DNA sequences. CRT works by first detecting repeats that are separated by a similar distance, and then checking for other CRISPR specific requirements (*e.g.*, the spacers need to be non-repeating and similarly sized). We modified CRT to consider incomplete repeats at the ends of contigs from whole-metagenome assembly, and call the modified program metaCRT.

### Identification of CRISPRs by similarity search

We implemented CRISPRAlign for identifying CRISPRs in a target sequence (a genome or a contig) that has repeats similar to a given CRISPR (query CRISPR). CRISPRAlign works by first detecting substrings in the target sequence (or its reverse complement) that are similar to the repeat sequence of a query CRISPR, and then checking for other requirements, as in metaCRT. Both metaCRT and CRISPRAlign are available for download at http://omics.informatics.indiana.edu/CRISPR/.

### Selection of known and novel CRISPRs for targeted assembly in HMP datasets

Using metaCRT and CRISPRAlign, we prepared a list of known CRISPRs repeats (identified from complete/draft bacterial genomes) and a list of potentially novel ones (identified only in the whole-metagenome assemblies from the HMP datasets) for further detailed study of their distributions among the HMP datasets. As we show in Results, the targeted assembly strategy is important for an efficient and comprehensive characterization of these CRISPRs in human microbiome datasets.

Known CRISPRs were first identified from the bacterial genomes (or drafts) collected in the IMG dataset (version 3.3), using metaCRT. We then selected a subset of the identified CRISPRs that meet the following requirements: direct repeats are of length 24–40 bps, there are a minimum of 4 copies of the direct repeats, and the individual repeats each differ by at most one nucleotide from the repeat consensus sequence, on average. The parameters were chosen to minimize false CRISPRs, considering that a CRISPR array typically contains 27 repeats, with an average repeat length of 32 base pairs [28]. We only kept CRISPRs that can be found in at least one of the whole-metagenome assemblies, using CRISPRAlign. We further reduced the number of candidate CRISPRs by keeping only those that share at most 90% sequence identity along their repeats by CD-HIT [39], as there are CRISPRs that share very similar repeats, and our targeted assembly strategy can recover the CRISPRs with slight repeat differences. To avoid including a repeat and its reverse complete (metaCRT does not consider the orientation for the repeats) in the non-redundant list, we included reverse complement sequences of the CRISPR repeats in the clustering process. Therefore, a repeat would be classified into two clusters by CD-HIT (the reverse complete of the repeat would be classified into a different cluster), one of which was removed to reduce redundancy.

We consider that a CRISPR identified in the HMP assemblies is novel if we find no instances of this CRISPR in the IMG bacterial genomes and the HMP reference genomes, with at most 4 mismatches using CRISPRAlign. Similarly, we only kept a non-redundant list of the novel candidates.

In total, we selected a collection of non-redundant CRISPRs—including 64 known CRISPRs and 86 novel ones—for further targeted assembly from HMP shotgun reads. The detailed information for these CRISPRs (repeat sequences, and their resources, and the references for the CRISPRs already collected in the CRISPRdb database http://crispr.u-psud.fr/ [6]), is provided in Tables S1 and S2.

### Targeted assembly of CRISPRs

For the targeted assembly of CRISPRs, we first carried out a BLASTN search with each putative CRISPR repeat sequence as the query, to collect reads that contain the repeat sequence (see Figure 1). In order to make the similarity search tolerant to sequencing errors and genomic variations that are observed among the multiple copies of a CRISPR repeat (in one CRISPR locus or between different CRISPR loci), we allowed three mismatches over the entire CRISPR repeat sequence: we retained only the reads that are aligned with the entire CRISPR repeat sequence with a maximum of three mismatches. With these reads containing CRISPR repeat sequences, we ran SOAPdenovo [40] with k-mers of 45 bps, which are sufficiently long to assemble reads with the repetitive sequences found in CRISPRs. In general, whole-metagenome contigs are assembled using shorter k-mers (for example, 21–23 bps in MetaHit [41] and 25 bps in HMP assembly [42]), as longer k-mers often fragment assemblies into short contigs. After CRISPR contigs were assembled, the exact boundaries of the repeats and spacers were obtained using CRISPRAlign.

### Validation of the targeted assembly approach using simulated datasets

We simulated short reads from 6 reference genomes (*Azospirillum B510*, *Streptococcus mutans NN2025*, *Deferribacter desulfuricans SSM1*, *Dehalococcoides GT*, *Erwinia amylovora ATCC 49946*, and *Escherichia coli K12 MG1655*), and applied our method to attempt to assemble the 10 known CRISPRs in these genomes. All 54 contigs assembled by our targeted assembly approach match perfectly to known CRISPRs in the reference genomes. We listed the genome names, the CRISPR repeats, the coordinates of the known CRISPRS in the reference genomes, and the coordinates of the contigs aligned on the reference genomes in Table S7.

### Datasets

We used the dataset Human Microbiome Illumina WGS Reads (HMIGWS) Build 1.0 available at http://hmpdacc.org/HMIWGS, and the whole-metagenome assemblies from the HMP consortium (http://www.hmpdacc.org/). The bacterial genomes were downloaded from the IMG database (http://img.jgi.doe.gov/cgi-bin/m/main.cgi), NCBI ftp site (ftp://ftp.ncbi.nih.gov/genomes), and human microbiome project website (http://www.hmpdacc.org/data_genomes.php). The viral genomes were downloaded from the NCBI ftp site (http://www.ncbi.nlm.nih.gov/genomes/GenomesGroup.cgi?taxid=10239). Additional phage genomes were downloaded from the PhAnToMe database site (http://www.phantome.org/Downloads/DNA/all_sequences/).

## Supporting Information

Figure S1 A network of 150 CRISPRS. The CRISPR names were shown in each node. The CRISPR host species for each known CRIPRS are listed in Table S2. Known CRISPRs are shown as blue nodes (except for several CRISPRs highlighted in green), and the novel CRISPRs identified in the HMP datasets are shown as red nodes.
(TIF)

Figure S2 The consensus of CRISPR repeats for 6 large clusters. See cluster ID in Figure S1. The sequence logo was prepared using weblogo (http://weblogo.berkeley.edu/).
(TIF)

Figure S3 Distribution of CRISPRs in different body sites. The x-axis represents 150 CRISPRs (listed in Table S2) and y-axis represents the proportion of samples in which instances of each of the CRISPR are found.
(TIF)

Figure S4 Cluster of spacers shared by more than eight samples. In this map, rows are spacers (clustered at 80% identify), and the columns are samples: cluster (a) is shared by 22 samples; cluster (b) is shared by 23 samples; cluster (c) is shared by 12 samples; cluster (d) is shared by 32 samples. The red lines indicate the presence of spacers in each of the samples. Multiple lines in the same row represent a spacer that is shared by multiple samples.
(TIF)

Figure S5 Sharing of streptococcal CRISPR spacers among samples from 6 individuals. In this map, the rows are the 761 spacers (clustered at 80% identify; see Figure 5 for the plot using 98% identify) identified in one or more of these 6 individuals, and the columns are samples (e.g., Stool_v1_p1 means a sample from stool of individual 1, in visit 1; Tongue_v2_p1 indicates dataset from tongue, individual 1, in visit 2). Buccal stands for buccal mucosa, and SupraPlaque stands for supragingival plaque. The red lines indicate the presence of spacers in each of the samples. Multiple lines in the same row represent a spacer that is shared by multiple samples.
(TIF)

Figure S6 Sharing of KoralL32 CRISPR spacers among samples from 6 individuals. In this map, rows are the 598 spacers (clustered at 80% identify), and the columns are samples (e.g., Stool_v1_p1 means a sample from stool of individual 1, in visit 1; tongue_v2_p1 indicates dataset from tongue, individual 1, in visit 2). The red lines indicate the presence of spacers in each of the samples. Multiple lines in the same row represent a spacer that is shared by multiple samples.
(TIF)

Figure S7 Sequence logos showing the short sequence motifs in regions adjacent to proto-spacers in the viral genomes for three CRISPRs.
(TIF)

Table S1 List of 150 CRISPRs studied in this manuscript and the targeted assembly results in the HMP datasets.
(DOCX)

Table S2 List of CRISPRs that are identified from the reference genomes, and their cross-references in the CRISPRdb.
(DOCX)

Table S3 List of numbers of datasets from different body sites that have reads (the first number) or CRISPRs (the second number) identified for each CRISPR.
(XLSX)

Table S4 List of viral genomes and their accession IDs plotted in Figure 6A.
(DOCX)

Table S5 List of viral genomes sharing high sequence similarities (≥90% identify over 30 bps) with CRISPR spacers.
(DOCX)

Table S6 List of plasmids sharing high sequence similarities (≥90%) with CRISPR spacers.
(DOCX)

Table S7 Targeted assembly results of 10 CRISPRs using reads simulated from 6 genomes.
(DOCX)
