# Supplementary figures and images for: Diverse CRISPRs Evolving in Human Microbiomes
Source: PLoS Genet. 2012 Jun 13;8(6):e1002441. doi: 10.1371/journal.pgen.1002441 (PMC3374615; doi:10.1371/journal.pgen.1002441)

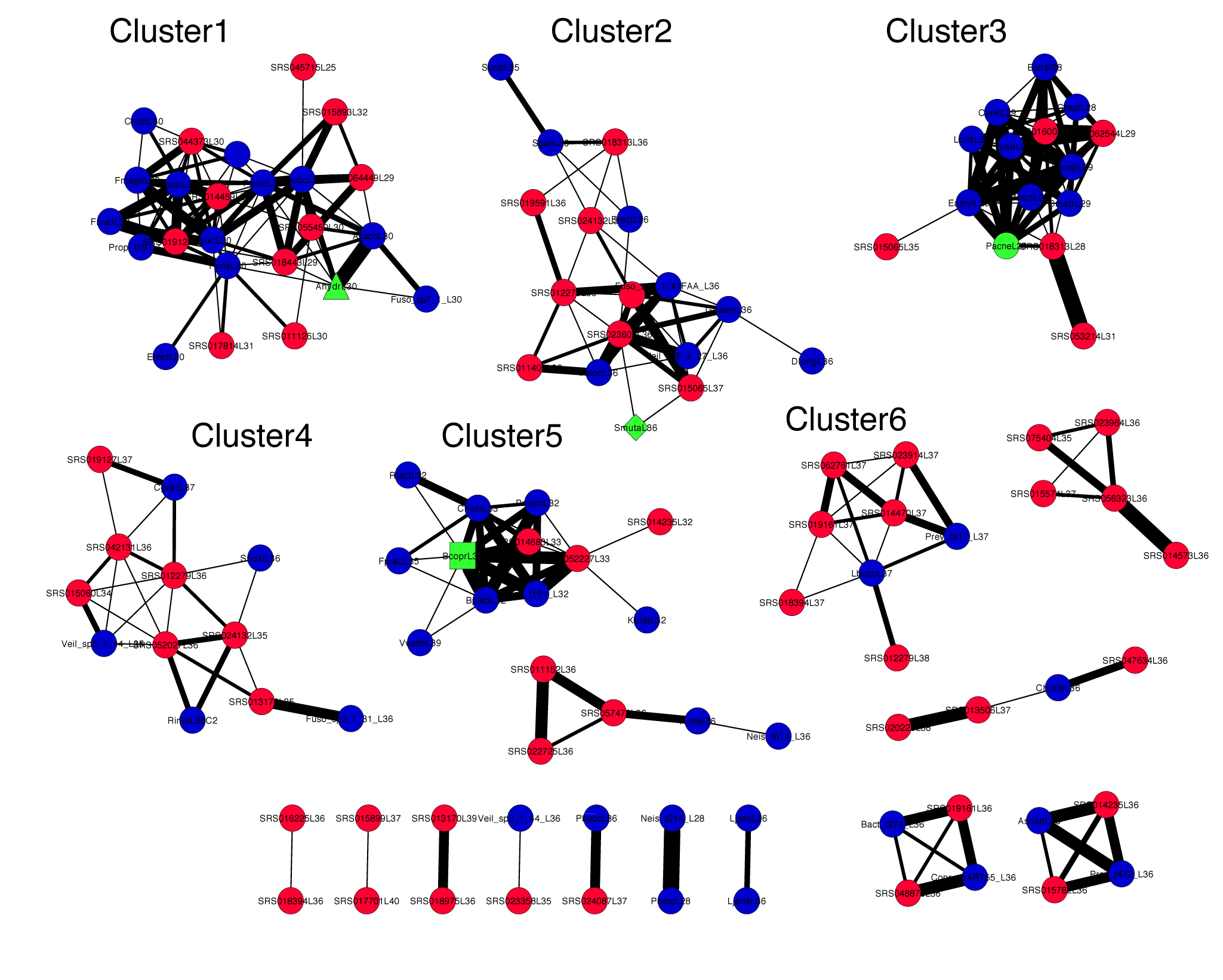

Supplement: Figure S1 — A network of 150 CRISPRS. The CRISPR names were shown in each node. The CRISPR host species for each known CRIPRS are listed in Table S2. Known CRISPRs are shown as blue nodes (except for several CRISPRs highlighted in green), and the novel CRISPRs identified in the HMP datasets are shown as red nodes. (TIF) [file pgen.1002441.s001.tif]

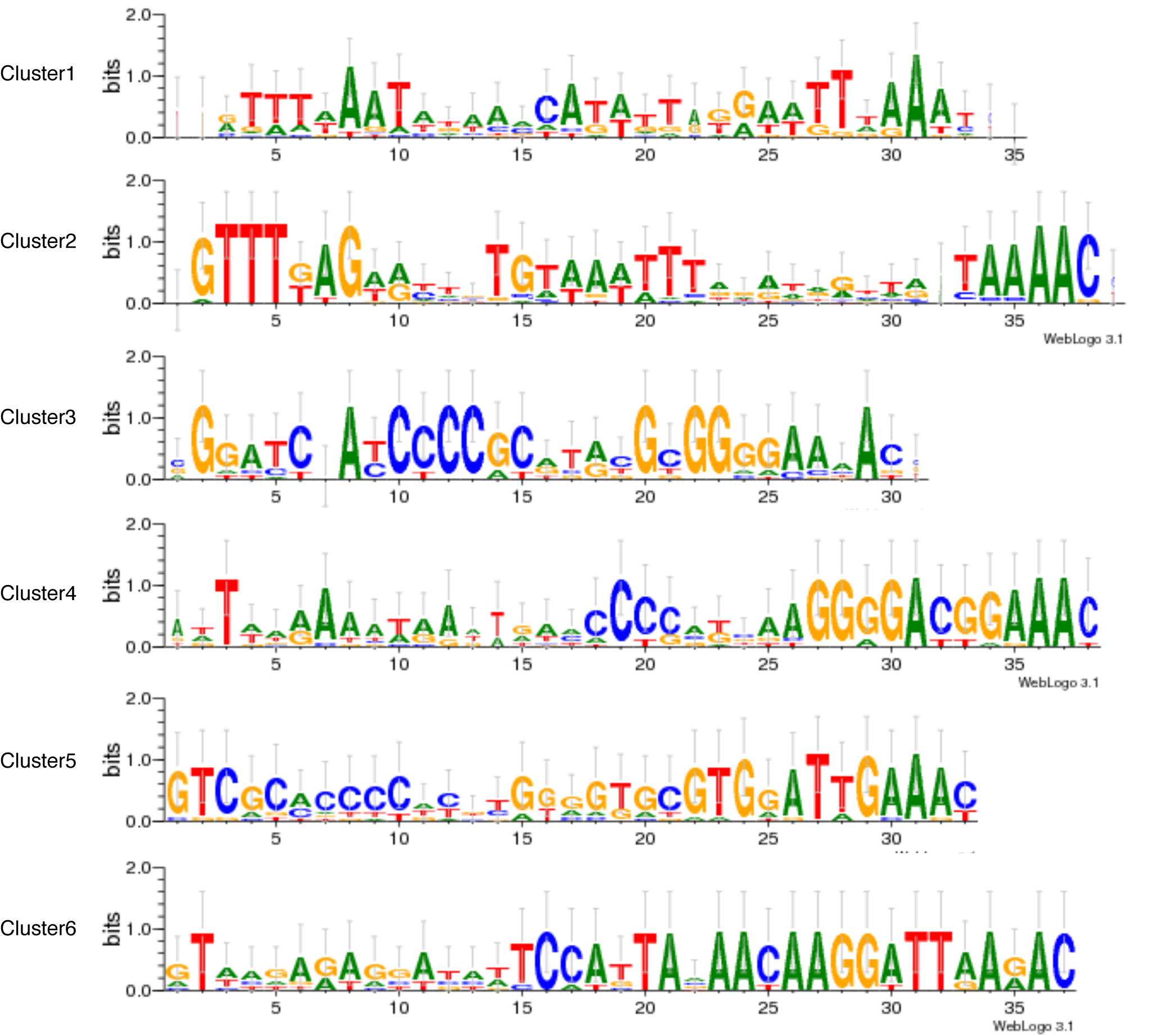

Supplement: Figure S2 — The consensus of CRISPR repeats for 6 large clusters. See cluster ID in Figure S1. The sequence logo was prepared using weblogo (http://weblogo.berkeley.edu/). (TIF) [file pgen.1002441.s002.tif]

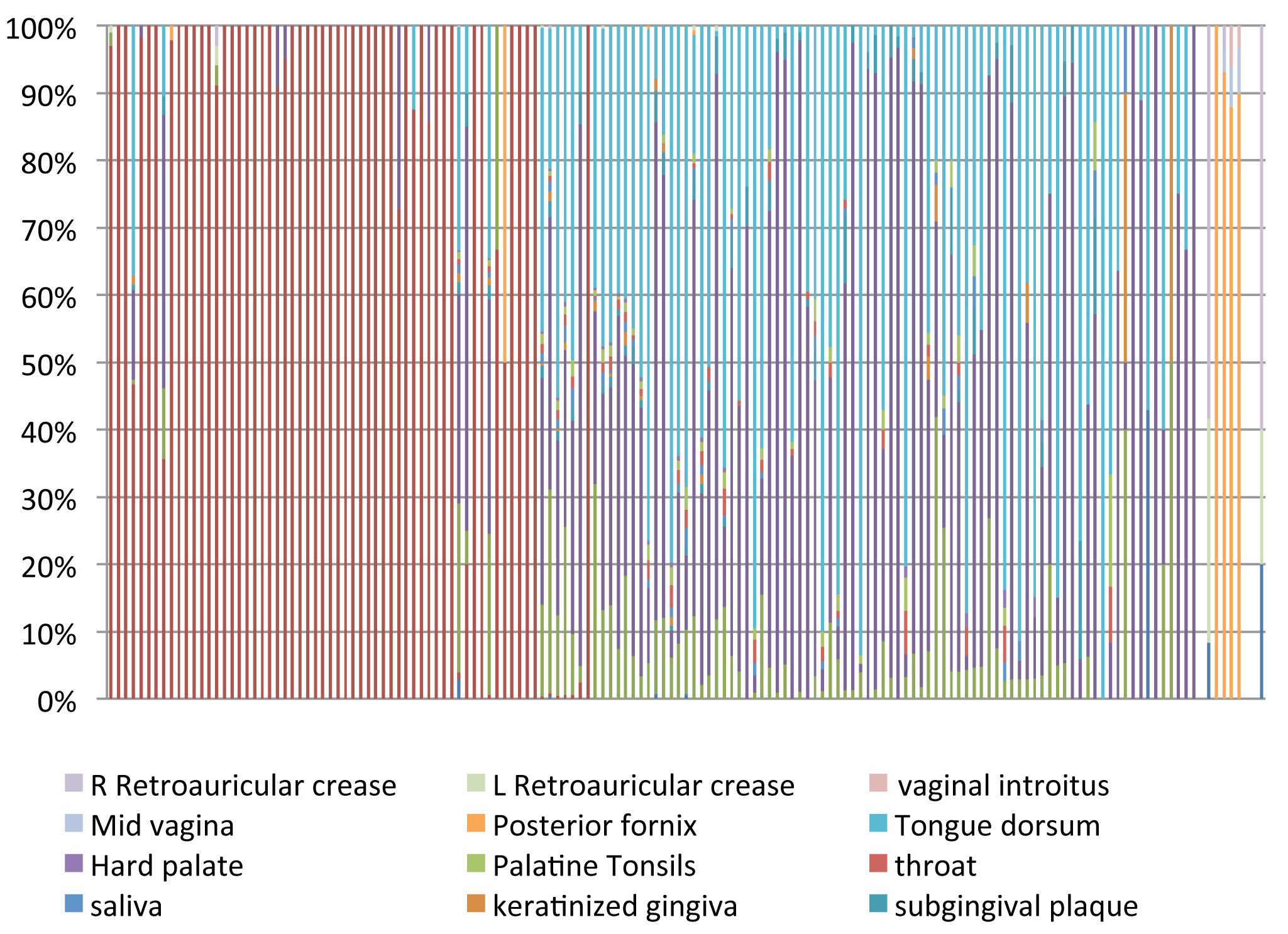

Supplement: Figure S3 — Distribution of CRISPRs in different body sites. The x-axis represents 150 CRISPRs (listed in Table S2) and y-axis represents the proportion of samples in which instances of each of the CRISPR are found. (TIF) [file pgen.1002441.s003.tif]

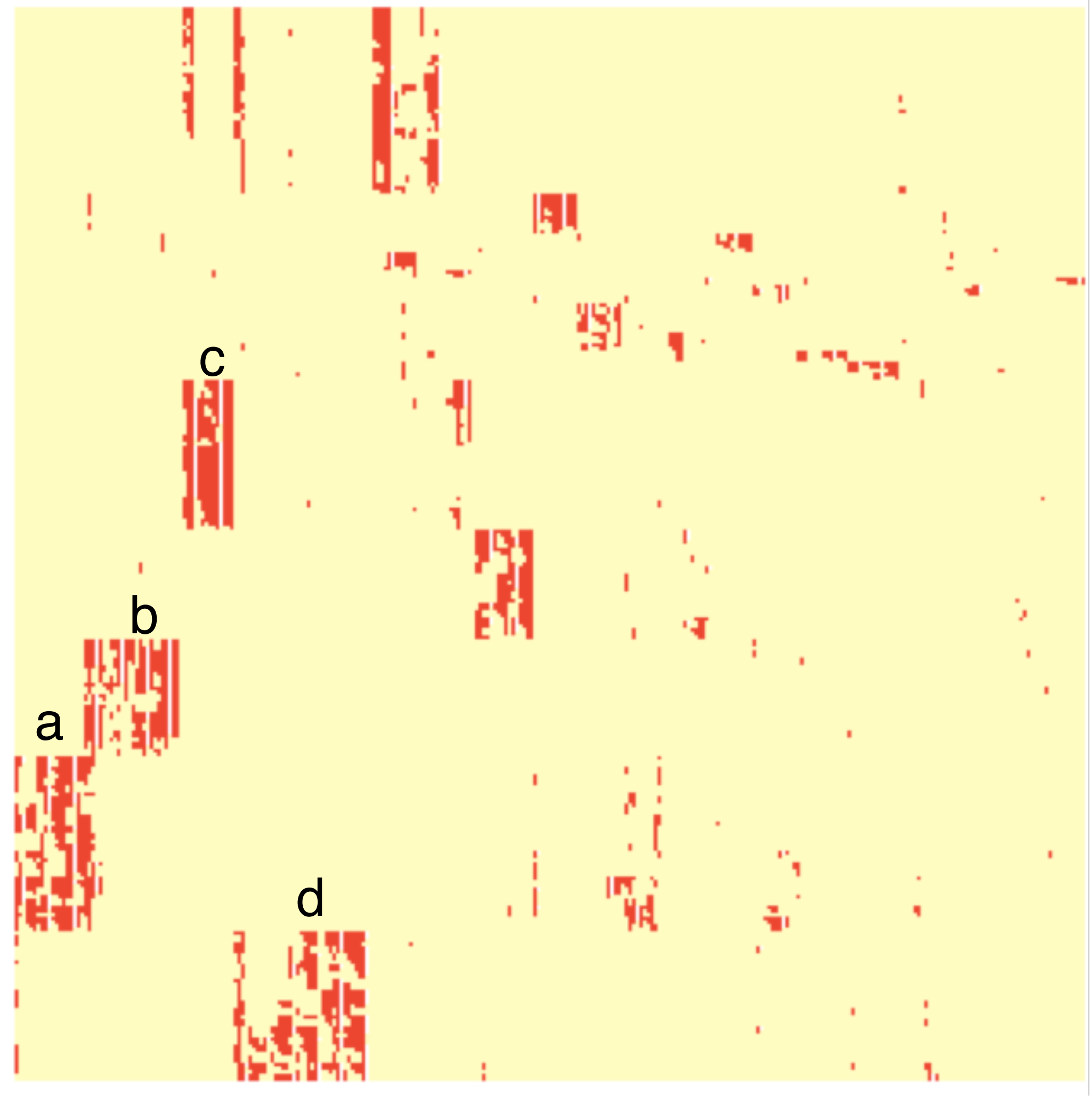

Supplement: Figure S4 — Cluster of spacers shared by more than eight samples. In this map, rows are spacers (clustered at 80% identify), and the columns are samples: cluster (a) is shared by 22 samples; cluster (b) is shared by 23 samples; cluster (c) is shared by 12 samples; cluster (d) is shared by 32 samples. The red lines indicate the presence of spacers in each of the samples. Multiple lines in the same row represent a spacer that is shared by multiple samples. (TIF) [file pgen.1002441.s004.tif]

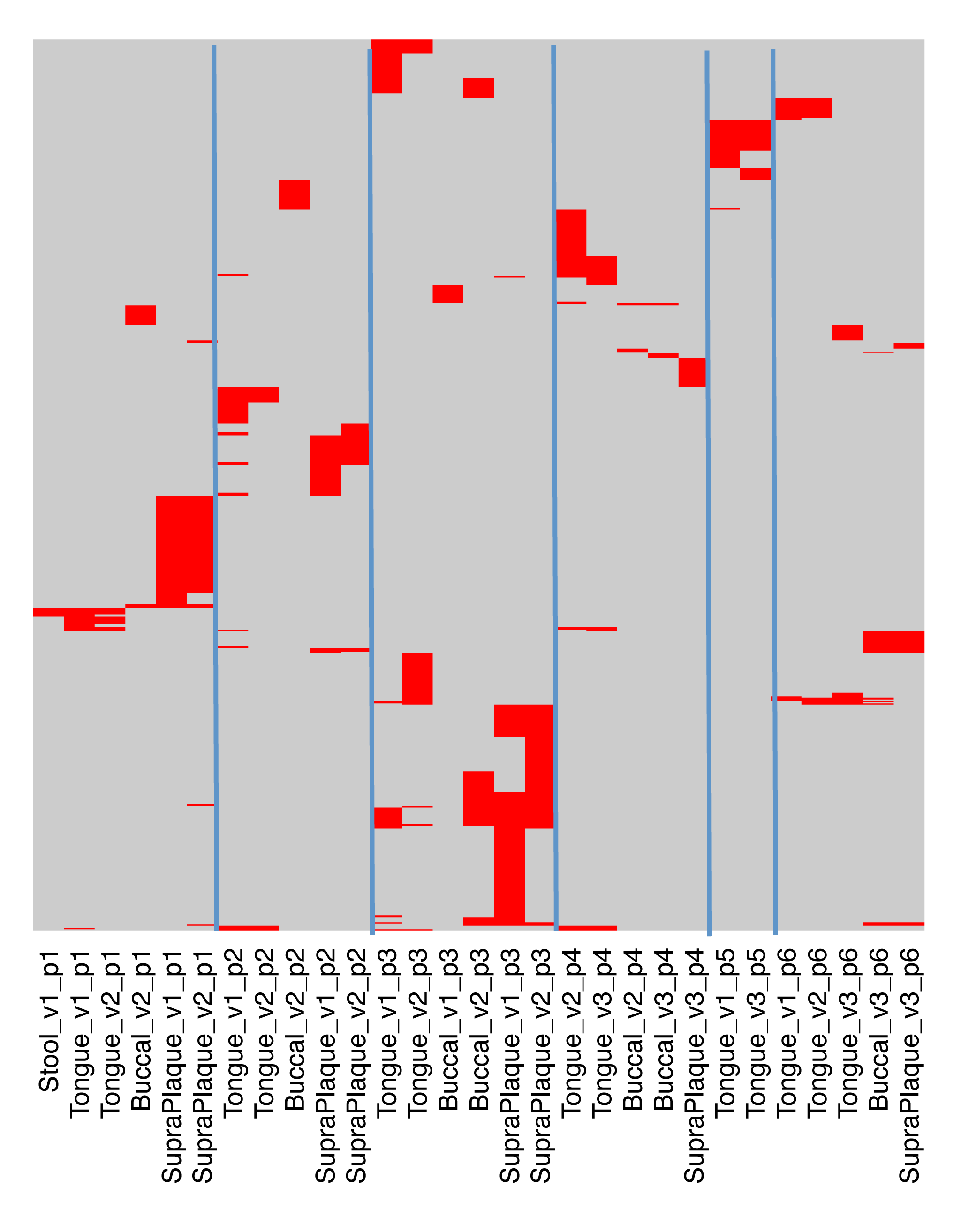

Supplement: Figure S5 — Sharing of streptococcal CRISPR spacers among samples from 6 individuals. In this map, the rows are the 761 spacers (clustered at 80% identify; see Figure 5 for the plot using 98% identify) identified in one or more of these 6 individuals, and the columns are samples (e.g., Stool_v1_p1 means a sample from stool of individual 1, in visit 1; Tongue_v2_p1 indicates dataset from tongue, individual 1, in visit 2). Buccal stands for buccal mucosa, and SupraPlaque stands for supragingival plaque. The red lines indicate the presence of spacers in each of the samples. Multiple lines in the same row represent a spacer that is shared by multiple samples. (TIF) [file pgen.1002441.s005.tif]

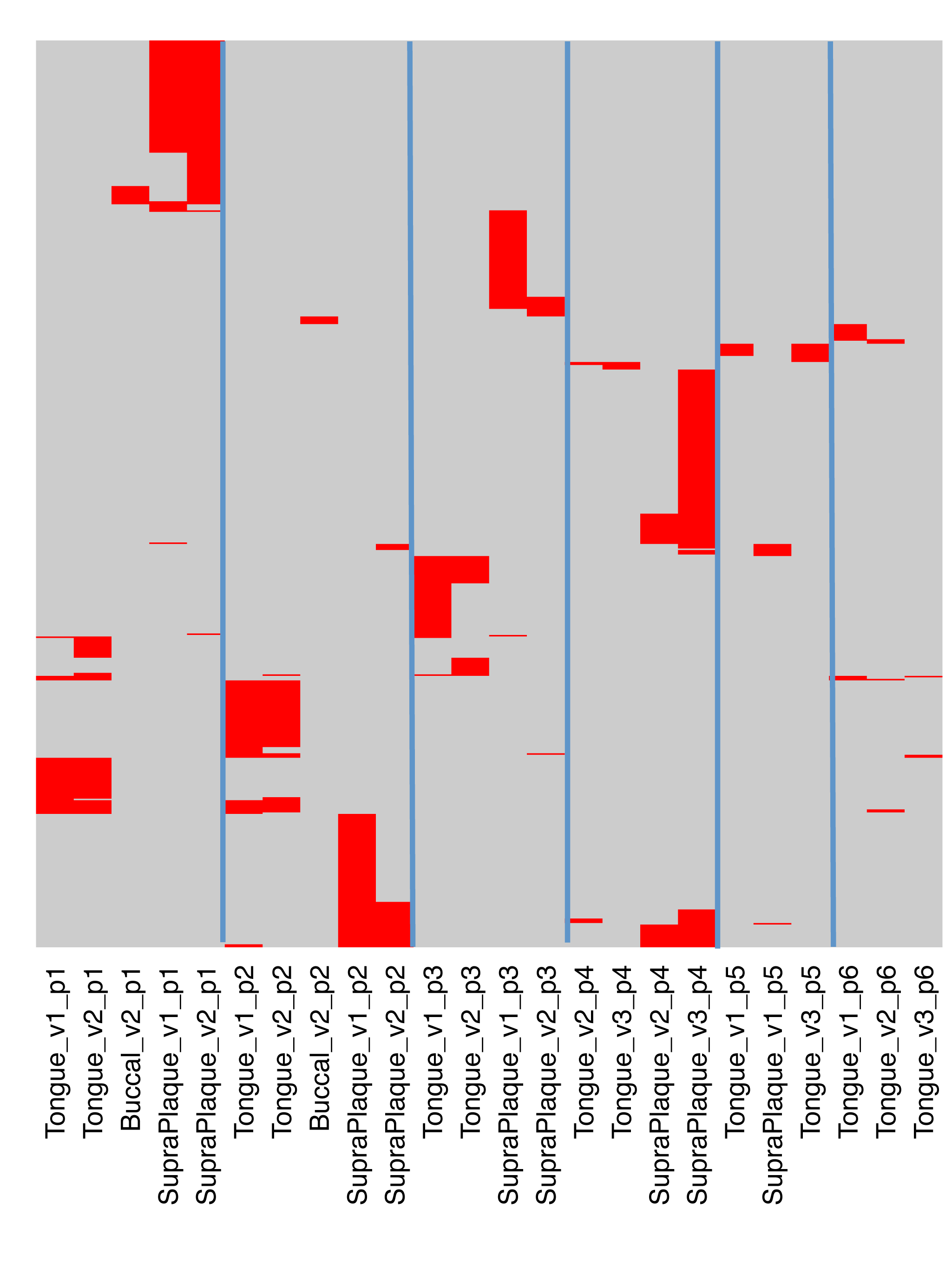

Supplement: Figure S6 — Sharing of KoralL32 CRISPR spacers among samples from 6 individuals. In this map, rows are the 598 spacers (clustered at 80% identify), and the columns are samples (e.g., Stool_v1_p1 means a sample from stool of individual 1, in visit 1; tongue_v2_p1 indicates dataset from tongue, individual 1, in visit 2). The red lines indicate the presence of spacers in each of the samples. Multiple lines in the same row represent a spacer that is shared by multiple samples. (TIF) [file pgen.1002441.s006.tif]

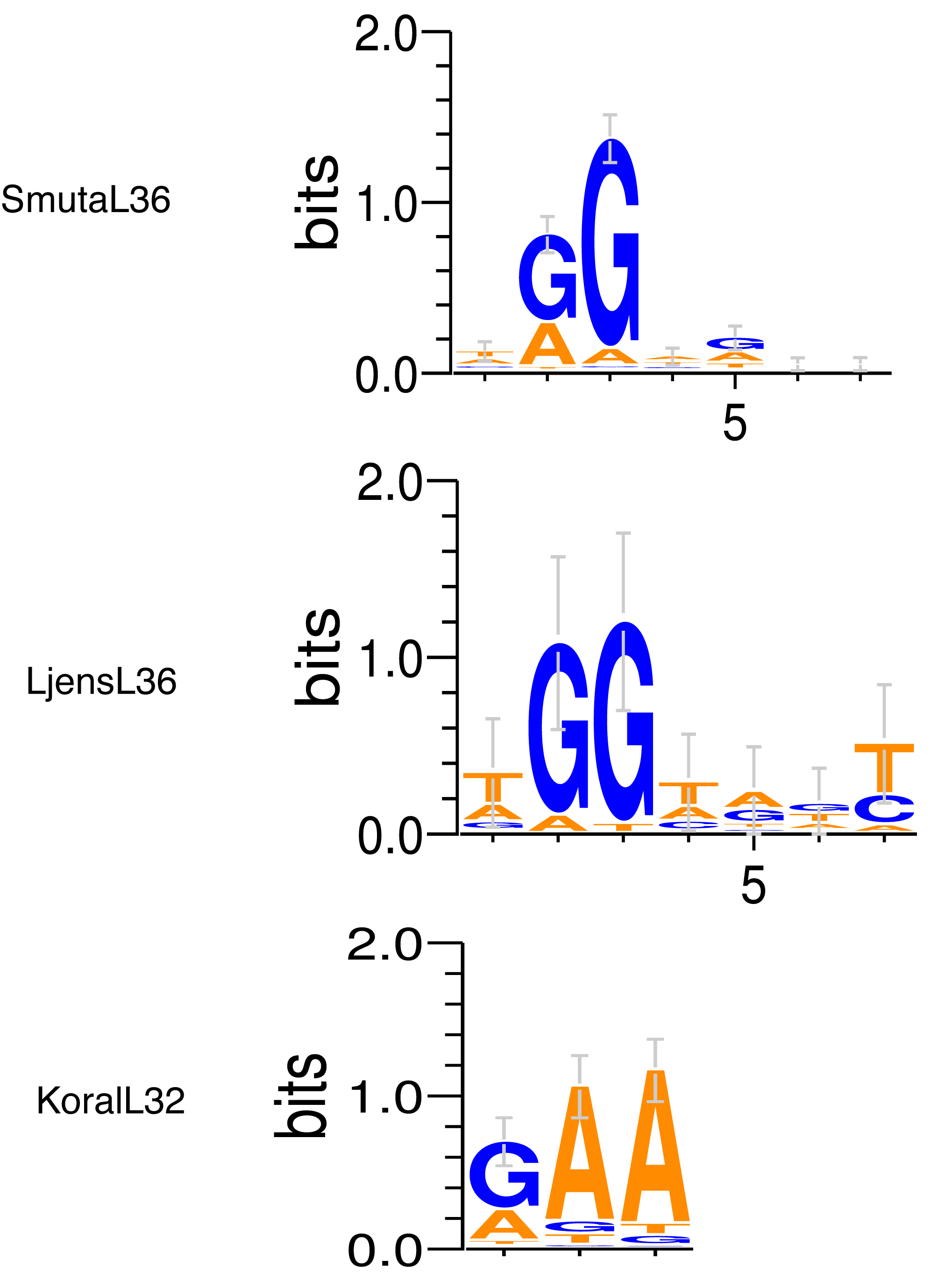

Supplement: Figure S7 — Sequence logos showing the short sequence motifs in regions adjacent to proto-spacers in the viral genomes for three CRISPRs. (TIF) [file pgen.1002441.s007.tif]
