# Supplementary material for: Diverse CRISPRs Evolving in Human Microbiomes
Source: PLoS Genet. 2012 Jun 13;8(6):e1002441. doi: 10.1371/journal.pgen.1002441 (PMC3374615; doi:10.1371/journal.pgen.1002441)
Supplement: Table S1 — List of 150 CRISPRs studied in this manuscript and the targeted assembly results in the HMP datasets. (DOCX) [file pgen.1002441.s008.docx]

Table S1. List of 150 CRISPRs studied in this manuscript and the targeted assembly results in the HMP datasets.

| ID^a^ | Consensus sequence of the CRISPR repeats | Number of HMP datasets with CRISPR identified | | Var^c^ | Mis^d^ |
| --- | --- | --- | --- | --- | --- |
|  |  | Whole-metagenome assembly^b^ | Targeted Assembly |  |  |
| AcaccL30 | ATTTCAATACATCTAATGTTTTTATTAATC | 4 | 44 | 10 | 2.5 |
| AhydrL30 | ATTTCAATACATCTAATGTTATTAATCAAC | 7 | 53 | 7 | 3 |
| AlactL29 | AGGATCATCCCCGCTTGTGCGGGTACAAC | 2 | 3 | 6 | 2.3 |
| AshahL36 | GTTGTGGTTTGATGTAGAATTTCGATAAGATACAAC | 23 | 99 | 12 | 1.5 |
| Bact_t274_L36 | GTCTATAAGACATTTATAATTTCTACTATTGTAGAT | 8 | 41 | 17 | 1.8 |
| BcoprL32 | GTCGCACCCTGCGTGGGTGCGTGGATTGAAAC | 25 | 65 | 13 | 2.2 |
| BplebL32 | GTTTCAATCCACGCACCTACGTGAGGTGCGAC | 3 | 22 | 14 | 2.5 |
| CgracL37 | GTTTGATTAGAATTTCCCCGATCTAGGGGATTGAAAC | 5 | 2 | 1 | 0 |
| ChomiL36 | GTCGCAATCCCTTTGGGTCAGGGACAGGTTTTTAAT | 11 | 77 | 30 | 1.1 |
| CjeikL29 | GTGCTCCCCGCGCCAGCGGGGATGAGCCG | 8 | 14 | 9 | 2.3 |
| CmatrL29 | GTATTCTCCGCGCACGCGGAGGTAGTTCC | 69 | 154 | 82 | 1.9 |
| CnexiL33 | GTCGCTCCCTTCACGGGGAGCGTGGATTGAAAT | 2 | 52 | 21 | 2.4 |
| Copr_spART55_L36 | ATCTACAACAGTAGAAATTATTTAAGGTTACTCAAC | 4 | 15 | 8 | 0.9 |
| CrectL30 | GTTTCAAACTCCAACGGAGTAAATTTTAAC | 23 | 109 | 36 | 1.6 |
| CtepiL28 | GTCTTCCCCACGCCCGTGGGGGTGTTTC | 1 | 11 | 6 | 1.3 |
| DlongL36 | GCTTGTGTACCATACATTTTTACATCATTCTCAAAC | 3 | 26 | 5 | 1.6 |
| EamylL29 | GTGTTCCCCGCGTATGCGGGGATAAACCG | 5 | 20 | 11 | 2.4 |
| ErectL30 | GTGTCAATTCCTTATAGGTAATGTATATCA | 2 | 5 | 4 | 0.7 |
| ErectL36 | ATTTTAGTAACTGAATAATTTACGTGACTGTAAAAC | 2 | 9 | 5 | 1.2 |
| EsiraL28 | CTTGTCCCCACACGCGTGGGGATGATCC | 4 | 14 | 19 | 1.6 |
| EsiraL32 | GTTGCAATCTACATGCACGGGTATAGTGCAAG | 1 | 2 | 3 | 0.7 |
| EyuriL30 | ATTTACATTCCAATCTGGTGATATTCAAAT | 13 | 121 | 45 | 1.6 |
| FalocL36 | GTTTGAGAGTAGTGTAATTTCATATGGTAGTCAAAC | 3 | 135 | 29 | 2.1 |
| FmagnL30 | ATTAAAATCTACCCAGAGTGGAATGTAAAT | 2 | 5 | 8 | 2.2 |
| FnuclL30 | ATTTAAATTCTAATATAGAAATACATAAAT | 4 | 149 | 22 | 1.4 |
| FperiL30 | CTTAAAGTATTATCATATTGGAATTTAAAT | 22 | 125 | 21 | 1.1 |
| FprauL29 | CCTTTTCCCCGCATACGCGGGGGTGATCC | 24 | 44 | 27 | 1.9 |
| FprauL35 | GTCGCCCTCCTCGCGGAGGGCGTGGATAGAAATAA | 2 | 19 | 5 | 1.4 |
| Fuso_sp1_1_41FAA_L36 | GTTTTATATCTATCTAAAATAACATTACTCTCAAAC | 6 | 110 | 8 | 1 |
| Fuso_sp2_1_31_L36 | ATAAGAATGAATGATACTCCGTAAGGAGACGGAAAT | 42 | 61 | 19 | 1.8 |
| Fuso_sp7_1_L30 | ATGAACTAAAAACTTGAAAAGTTTTGAAAT | 10 | 147 | 31 | 1.9 |
| GhaemL36 | GTTTGAGAGATATGTAAATTTTGAATTCTACAAAAC | 8 | 257 | 57 | 1.5 |
| KoralL32 | GTTTCAACACACAGCCGCCCGAAGGCGGCTGC | 65 | 339 | 136 | 2.1 |
| LbuccL29 | GTTTTAATAGCACAAATTGTATTGTAAAT | 2 | 40 | 7 | 0.8 |
| LbuccL37 | GTTTCAATCCTTGTTTTAATGGATACTCTACTTTAAC | 22 | 178 | 34 | 1.9 |
| LcrisL29 | AGGATCACCTCCACATACGTGGAGAATAC | 24 | 30 | 8 | 1.6 |
| LjassL36 | GTTTTAGATGGTTGTTAGATCAATAAGGTTTAGATC | 8 | 33 | 3 | 1.3 |
| LjensL36 | GTTTTAGAAGGTTGTTAAATCAGTAAGTTGAAAAAC | 4 | 29 | 2 | 0.5 |
| MhypeL30 | ATTTAACTTTAACAAGAGTTGTATTTGAAT | 2 | 8 | 4 | 1.2 |
| Neis_t014_L28 | GTTACCTGCCGCACAGGCAGCTTAGAAA | 13 | 142 | 20 | 2.7 |
| Neis_t014_L36 | GTTGTAGCTCCCTTTCTCATTTCGCAGTGCTACAAT | 44 | 202 | 15 | 1 |
| PacneL29 | GTATTCCCCGCCTATGCGGGGGTGAGCCC | 1 | 12 | 5 | 0.8 |
| PbuccL36 | GTTGCATCTGCCTTCTTTTTGAAAGGTAAAAACAAC | 1 | 50 | 10 | 2.6 |
| PcaroL28 | GTTCACTGCCGCATAGGCAGCTTAGAAA | 13 | 11 | 4 | 2.2 |
| PmerdL32 | GTCGTACCTTATATAGGTACGTGGATTGAAAC | 12 | 58 | 10 | 1.6 |
| PmicrL30 | GTTTAAATAGAAACATACTGTAATGTAAAT | 5 | 50 | 20 | 1.1 |
| PorisL30 | GTTATAATTGAACCTTATTGGAATTGAAAC | 11 | 91 | 12 | 1.3 |
| PpropL29 | CGGTTCATCCCCGCGCATGCGGGGAACAC | 5 | 11 | 7 | 3.7 |
| Prev_t317_L37 | GTACGAGTGCACCATCCATTAAAACAAGGATTAAGAC | 4 | 81 | 14 | 2.4 |
| Prev_t472_L36 | GTTGTATTATATCTTCATTCTACATCAAACCACAAC | 3 | 125 | 18 | 1.6 |
| Prop_t191_L30 | CTCCAAATTGTACCTTAGTGGAATTGAAAT | 7 | 43 | 5 | 0.8 |
| Prop_t191_L32 | GTTTCAATTCACGCACCCCTAAAGGGTGCGAC | 1 | 14 | 4 | 1.0 |
| RdentL36 | CCCTCAATGAAAGTCACCCATTCTCATGGGTGAGAC | 9 | 60 | 29 | 1.7 |
| RinteL36 | ATTATACCATACCAAGTGATAACAGGGAATTACAAC | 3 | 36 | 2 | 0.5 |
| RinteL36C2 | ATTGAGATAAGAGAACCCCGTGGAGGGGACGAGAAC | 7 | 26 | 14 | 1.9 |
| RlactL32 | GTCACTTCCCTTGCGGAAGTGTGGATTGAAAT | 1 | 34 | 8 | 1.5 |
| SgallL36 | GTTTTACGGTTACTTAAATCTTGAGAGTACAAAAAC | 31 | 41 | 7 | 1.4 |
| SmutaL36 | GTTTTAGAGCTGTGTTGTTTCGAATGGTTCCAAAAC | 31 | 386 | 35 | 1.4 |
| SnoxiL36 | AGTTTCCGTCCCCTTGCGGGGATGTGGTTTTGAAAT | 5 | 34 | 6 | 1.3 |
| SoralL35 | GTTTTTGTAGTCTCCAAAATTGTGACCGATAAAAC | 10 | 264 | 23 | 1.2 |
| Veil_sp3_1_44_L35 | GTTAAATTAAATAATCCCCTGATGGGGACGGAAAC | 148 | 271 | 71 | 2.6 |
| Veil_sp3_1_44_L36 | GTTGCAACAGTACTTACCGTTTACGGTATTGAAACT | 87 | 219 | 17 | 1.6 |
| Veil_sp6_1_27_L36 | GTTTGAGAGTAGTGTAATTCTGTAAACCTCTAAAAC | 46 | 258 | 5 | 1.4 |
| VvadeL39 | GTCCCCCTCCAACCGTAGTGGATTAGAAAC | 1 | 3 | 6 | 1.8 |
| SRS011126L30 | GTTTTAAATAAAGTATTGGAAGTTTAGAAC | 17 | 142 | 33 | 1.4 |
| SRS011152L36 | GTTATAGTTCCCTAACAATTTAGATATTGGTATAAT | 26 | 189 | 14 | 1.6 |
| SRS011405L36 | GTTTGAGTATGGTGCAATTTTAAAGGGTAGTCAAAC | 5 | 28 | 7 | 1.4 |
| SRS012273L36 | GTTTTAGTATTGTGTTATTTTAGATAGTAATAAAAC | 3 | 15 | 7 | 1.4 |
| SRS012279L36 | GTTTCCGTCCCCTAGACGGGGAATCATTTTCTAAAC | 24 | 84 | 45 | 2.1 |
| SRS012279L38 | TATAAAAGAAGAGAATCCAGTAGAATAAGGATTGAAAC | 16 | 97 | 20 | 1.3 |
| SRS013170L35 | TTTCCGTCTCCTCTCGGAGTTCACTCATTTCTTAT | 2 | 9 | 26 | 1.6 |
| SRS013170L39 | ATTGTTCCGGTCTTCCAGGCCGGACTACATTGAGACGAC | 3 | 18 | 2 | 1.5 |
| SRS013506L37 | GTCTCAATCCCTTTGGAACAGGGCAATGTCTTTCGAC | 15 | 168 | 127 | 3.1 |
| SRS013946L30 | AAGTTACTTAAATTGAAAGCTAATTACAAC | 9 | 55 | 2 | 0.5 |
| SRS013949L37 | GTTTCCATCCGCTATATCGCGGTACTTGGTATCTGAC | 6 | 103 | 9 | 1.8 |
| SRS013951L35 | GTTATAAGGAAGCAATAAATGCCTGCTGATGCAGG | 3 | 7 | 5 | 1 |
| SRS014124L25 | GGGTGTTTAATGTTGGGTGTTGATG | 87 | 170 | 15 | 1.3 |
| SRS014235L32 | GTTGCAATCCTCGTAGACAAGTGGGTTGCCAG | 3 | 9 | 24 | 2.2 |
| SRS014235L36 | GTTGTGGTTTGATGTAGAAACTGAATAGGATACAAC | 8 | 20 | 60 | 1.5 |
| SRS014235L37 | GTGTCAATGCCCTGAAAGGGGCGACCTTCATTTCTAC | 6 | 12 | 17 | 2.1 |
| SRS014459L30 | CTATAAATCTATCCATATTGGATTTTAAAT | 6 | 14 | 5 | 1 |
| SRS014470L24 | TATAGATTTAGTCCATAGGACTTT | 23 | 37 | 3 | 1.3 |
| SRS014470L37 | GTCTTAATCCTTGTTTTAATGGAAGATACTCTCAGAG | 39 | 146 | 2 | 1 |
| SRS014573L36 | CAGTATATCAAAGGGGATGGGTGATCAATTCTCAGC | 4 | 47 | 3 | 1 |
| SRS014683L33 | ACTTCAATCCACGCCCCACATGCGTGGGGCGAC | 1 | 2 | 4 | 2.2 |
| SRS015060L34 | GATAAAAAATAACCCCCAATACGGGGACGGAAAC | 2 | 10 | 18 | 1.5 |
| SRS015065L35 | AGGTTTCAATTCCCGCAACGCGGGTAAGATACATC | 5 | 2 | 33 | 1.7 |
| SRS015065L36 | GTTGGTTTTATCATTGTTTTGAATGGTATATCCAAC | 1 | 5 | 6 | 1.3 |
| SRS015065L37 | AGTTTTGTACCTTTATGAATTAACAAAGCTCTCAAAC | 2 | 26 | 1 | 0 |
| SRS015133L36 | CTACTACACTGGTGCGAATTTGCACTAGTCTAAAAC | 28 | 64 | 26 | 1.9 |
| SRS015378L34 | CCTCAATGAAGTGCAGTTCCCGAAGGAACTGCAA | 23 | 98 | 20 | 1.6 |
| SRS015574L37 | CAAGTATATCAAGGGATTAGAGAGTTGAACCCCAGCA | 49 | 19 | 43 | 1.5 |
| SRS015782L36 | GTTATGGTTTGATGTAGGAACAAGATGATAAACAAC | 2 | 2 | 5 | 0.8 |
| SRS015893L32 | ATTTACATTACACTTTGCTACTATTAATACCA | 12 | 91 | 2 | 0.5 |
| SRS015899L37 | AATCACAAAGATACAAATTTTGAAAGTAATTCACAAC | 11 | 58 | 11 | 1.2 |
| SRS016002L28 | ATTTGTCCCGCGTATGCGGGGATGATCC | 17 | 114 | 12 | 1.7 |
| SRS016225L36 | CTCTGAAAAACCTCCCCACCACATGGGGATTAAGAC | 7 | 51 | 19 | 1.7 |
| SRS017307L37 | GCAGAGATCATAACGCTACGAGCTATAGCACTGAAAC | 6 | 21 | 30 | 1.2 |
| SRS017445L37 | GTCGCAATGGAGCCTGACCTTTGAGGTCAGGAAAAGG | 11 | 63 | 22 | 1.7 |
| SRS017701L40 | GTTGTGATTCGCTTTCAAATTTGTATCTTTGACATATTAA | 31 | 41 | 10 | 1.5 |
| SRS017814L31 | ATATTAATCGCACCAGTTAAGGAATTGAAAC | 19 | 88 | 5 | 1.2 |
| SRS018300L36 | ATTTTACTACCTGGGAGATCGTACGGTAATCAAGAC | 8 | 35 | 63 | 2.5 |
| SRS018313L28 | GTTTTCGCCCCCTTACGGGGATGGTACC | 18 | 25 | 2 | 0.5 |
| SRS018313L36 | GTTTTAGTACCTGGAAGAATTGAGTCATCATAAAAC | 1 | 18 | 76 | 2.3 |
| SRS018394L36 | ATTAAAAACAAAGCCCCATCACTAGGGGATTAAGAC | 4 | 20 | 60 | 2.1 |
| SRS018394L37 | GTATTGAAGGTCATCCATTTATAACAAGGTTTAAAAC | 22 | 238 | 2 | 0.5 |
| SRS018443L29 | ATTTAATAGGAACATTTTGTATTGTAAAG | 15 | 16 | 69 | 2.0 |
| SRS018443L37 | GTCAGGGAGCACCCAGCACCACCAGGTGCATTAAGAC | 8 | 71 | 2 | 0.5 |
| SRS018975L36 | GTCTCAATGAAGTCCGGCCTAGAAGGCCGGAACAAT | 9 | 94 | 1 | 0 |
| SRS019124L30 | ATTTATGTAAAACCATATTGGAATTTAAAT | 1 | 4 | 34 | 1.3 |
| SRS019127L37 | ATTTAAACAAATTTGACCCGATCAAGGGGATTGAAAC | 39 | 144 | 17 | 2.5 |
| SRS019161L36 | GTTTAATACCTTATATAAATTTCTACTATTGTAGAT | 3 | 11 | 10 | 1.1 |
| SRS019161L37 | GTTAGAAAGGTTATTCCAGTAGAACAAGGATTAAGAC | 3 | 6 | 26 | 1.5 |
| SRS019591L36 | GTTTTAGTACTCTGTAATTTTTCGCTATGATAAAAC | 48 | 167 | 3 | 1.0 |
| SRS020226L38 | AGTCGAAAGACACCGCCCTGCTCTAAAGGGATTGAGAC | 3 | 70 | 19 | 1.7 |
| SRS020869L32 | GTAACCGTCCCCTCGCGGGGCTAAGCTGCATC | 2 | 11 | 9 | 1.4 |
| SRS021496L35 | GTTTCGATGCTCAATGTTTTGAGCTTTTTCTTTAT | 12 | 20 | 3 | 0.7 |
| SRS022609L28 | GTTGTGATTTGATGGTTTTCAAAGAGGG | 1 | 3 | 3 | 1.3 |
| SRS022609L36 | GTTTCAATCACCTCGCGGTGAGGGGGTGGTTCAGAC | 8 | 25 | 11 | 1.6 |
| SRS022725L36 | ATTATACTACTATCGAAATTATTAGGGGACTATAAC | 3 | 29 | 28 | 1.7 |
| SRS023358L35 | GTTACACAGGCGAATACCGCTTGCGGTATTGAAAC | 1 | 10 | 7 | 2 |
| SRS023604L36 | GTTTGAGAGTAGTGTAATTTATGAAGGTACTAAAAC | 1 | 4 | 23 | 2.4 |
| SRS023914L37 | GTCTTAATCCTTGTTGTAGTGGACAATGGTCTCGTAC | 4 | 7 | 2 | 0.5 |
| SRS023964L36 | GAAGTCTATCAAGGGGTCTGGTGACTGAATCCCAGC | 11 | 127 | 7 | 1.3 |
| SRS024075L27 | AACCAGCTGACAGAACTGGATGTGAGC | 8 | 15 | 1` | 0 |
| SRS024087L24 | CGCGTTTCCTATTCATTAGATGAA | 28 | 42 | 4 | 2.5 |
| SRS024087L37 | GTTGTTTTTACCTCTCAAAAAGGAGGTAGACACAACC | 2 | 7 | 73 | 2.3 |
| SRS024132L35 | TTTCCGTCCCCTTCCCGGGGATCTTATTTCTCAAT | 1 | 9 | 10 | 0.9 |
| SRS024132L36 | GTTGTATATCTCTCAAATTATACAGGATCCTAAAAC | 1 | 3 | 15 | 2 |
| SRS024144L38 | GCATTATCCTTGTCTTGTGGCTTATCTGAAGGTGCTGG | 0 | 5 | 51 | 2.5 |
| SRS042131L36 | GTTTACAGAAGTTAACCCCGATAAGGGGACGGAAAC | 17 | 122 | 19 | 1.3 |
| SRS042984L36 | GTCTCTTCCGCTTATGCGGAAGACTTTCATTGAGGC | 7 | 78 | 6 | 0.8 |
| SRS044373L30 | ATTCAAATTTAGACAGACTGGAATTTAAAG | 3 | 17 | 3 | 1.7 |
| SRS045715L25 | GCTTTAAACTACGAATTTACGAATT | 59 | 77 | 48 | 1.6 |
| SRS047634L36 | GAATAAAAAACGTCCCTGAACCAAAGGGATTAAGAC | 11 | 64 | 10 | 0.9 |
| SRS047824L36 | GTTTGAGAGCTTTGTTAATTTGATAAGATATAAAAC | 2 | 33 | 3 | 0.7 |
| SRS048791L30 | TTAAGCTATGGTATATATACCATACCTTAA | 13 | 12 | 14 | 1.3 |
| SRS048791L36 | GTTTTAGTCCCCTTCGATATTGGGGTGGTCTATATC | 21 | 204 | 2 | 2.5 |
| SRS048870L36 | ATCTACAACAGTAGAAATTATTCTATATTACTTAAC | 11 | 47 | 7 | 1.1 |
| SRS052027L36 | ATTAGAAGATAGAGAACCCCAGTAGGGGACGGAAAC | 3 | 8 | 64 | 1.6 |
| SRS052227L33 | GTCGCAGCCCGCTTGGGCTGCGTGAATTGAAAC | 6 | 57 | 4 | 1.2 |
| SRS053214L31 | GCCGTACCATCCCCGTAAGGGGGCGAAAACC | 1 | 1 | 4 | 1.8 |
| SRS055450L30 | GTTTAAAACCCACATTTGATGTTTTAAAAC | 1 | 3 | 30 | 2.3 |
| SRS056323L36 | GCTGGGAATCAATCACCAATCCCCTTTGATATACTG | 25 | 147 | 11 | 1.1 |
| SRS057478L36 | GTTGTAGTTCCCTAACAGTTCTTGGTATGGTATAAT | 4 | 41 | 3 | 1 |
| SRS058808L26 | ATGTTCTCCGGAGCTACTGCATTCAA | 5 | 4 | 8 | 1.1 |
| SRS062544L29 | GGATCACCCCCGCTTGCGCGGGACAAATT | 4 | 90 | 156 | 2.0 |
| SRS062761L37 | GTCTTAATCCTTGTTGTAATGGAATATACTTAATAAT | 53 | 148 | 97 | 2.1 |
| SRS064449L29 | GTATTAATAGAATATTTTATAATGTAAAT | 1 | 35 | 67 | 1.7 |
| SRS075404L35 | AAGTGTATCAAAGGGGAATCACGATTGAATCCCAGC | 3 | 13 | 10 | 1.7 |

^a^: See Table 1 for naming conventions for the CRISPRs. ^b^:The “Whole-metagenome assembly” and “Targeted assembly” columns list the total number of samples in which each CRISPR was identified using the whole-metagenome assemblies and the targeted assembly approach, respectively. ^c^: the number of unique repeat sequences, and ^d^: the average mismatches between the unique repeats for all the repeats found in the HMP datasets for each CRISPR type using the targeted assembly approach.
