# Supplementary material for: Diverse CRISPRs Evolving in Human Microbiomes
Source: PLoS Genet. 2012 Jun 13;8(6):e1002441. doi: 10.1371/journal.pgen.1002441 (PMC3374615; doi:10.1371/journal.pgen.1002441)
Supplement: Table S2 — List of CRISPRs that are identified from the reference genomes, and their cross-references in the CRISPRdb. (DOCX) [file pgen.1002441.s009.docx]

Table S2. List of CRISPRs that are identified from the reference genomes, and their cross-references in the CRISPRdb.

| CRISPR ID | Reference genome | Number of repeats in the reference genome | | CRISPRdb ID |
| --- | --- | --- | --- | --- |
|  |  | Total | As the cons* |  |
| AcaccL30 | NZ_ABAX03000001 *Anaerostipes caccae* DSM 14662 | 13 | 11 |  |
| AhydrL30 | NZ_ABXA01000037 *Anaerococcus hydrogenalis* DSM 7454 | 12 | 10 |  |
| AlactL29 | NZ_ABYO01000191 *Anaerococcus lactolyticus* ATCC 51172 | 26 | 22 |  |
| AshahL36 | FP929032 *Alistipes shahii* WAL 8301 | 13 | 13 |  |
| Bact_t274_L36 | NZ_GG774890 *Bacteroidetes* oral taxon 274 str. F0058 | 11 | 11 |  |
| BcoprL32 | NZ_ACBW01000157 *Bacteroides coprophilus* DSM 18228 | 16 | 15 | NC_009615_1 |
| BplebL32 | NZ_ABQC02000012 *Bacteroides plebeius* DSM 17135 | 15 | 12 |  |
| CgracL37 | NZ_ACYG01000030 *Campylobacter gracilis* RM3268 | 21 | 14 |  |
| ChomiL36 | NZ_ACKY01000011 *Cardiobacterium hominis* ATCC 15826 | 32 | 30 |  |
| CjeikL29 | NZ_ACYW01000027 *Corynebacterium jeikeium* ATCC 43734 | 42 | 31 | NC_007164_2 |
| CmatrL29 | NZ_ACSH01000011 *Corynebacterium matruchotii* ATCC 14266 | 50 | 45 |  |
| CnexiL33 | NZ_ABWO01000146 *Clostridium nexile* DSM 1787 | 13 | 12 | NC_013216_10 |
| Copr_spART55_L36 | FP929039 *Coprococcus* sp. ART55/1 | 14 | 14 | NC_012778_1 |
| CrectL30 | NZ_ACFU01000001 *Campylobacter rectus* RM3267 | 32 | 31 |  |
| CtepiL28 | NC_002932 *Chlorobium tepidum* TLS | 19 | 19 | NC_011768_3 |
| DlongL36 | NZ_AAXB02000018 *Dorea longicatena* DSM 13814 | 20 | 19 |  |
| EamylL29 | NC_013961 *Erwinia amylovora* CFBP1430 | 33 | 30 | NC_012214_3 |
| ErectL30 | FP929043 *Eubacterium rectale* M104/1 |  |  |  |
| ErectL36 | NC_012781 *Eubacterium rectale* ATCC 33656 | 45 | 44 | NC_012781_2 |
| EsiraL28 | FP929059 *Eubacterium siraeum* V10Sc8a | 50 | 41 |  |
| EsiraL32 | NZ_ABCA03000040 *Eubacterium siraeum* DSM 15702 | 8 | 5 |  |
| EyuriL30 | GL405246 *Eubacterium yurii* subsp. margaretiae ATCC 43715 | 6 | 5 |  |
| FalocL36 | NZ_GG745527 *Filifactor alocis* ATCC 35896 | 23 | 22 |  |
| FmagnL30 | NZ_AECM01000022 *Finegoldia magna* ACS-171-V-Col3 | 88 | 43 |  |
| FnuclL30 | NC_003454 *Fusobacterium nucleatum* subsp. nucleatum ATCC 25586 | 30 | 28 | NC_003454_2 |
| FperiL30 | NZ_ACJY01000037 *Fusobacterium periodonticum* ATCC 33693 | 44 | 36 |  |
| FprauL29 | NZ_ABED02000028 *Faecalibacterium prausnitzii* M21/2 | 5 | 1 |  |
| FprauL35 | FP929045 *Faecalibacterium prausnitzii* L2/6 | 8 | 2 |  |
| Fuso_sp1_1_41FAA_L36 | NZ_GG770381 *Fusobacterium* sp. 1_1_41FAA | 24 | 23 |  |
| Fuso_sp2_1_31_L36 | NZ_GG657936 *Fusobacterium* sp. 2_1_31 | 11 | 11 |  |
| Fuso_sp7_1_L30 | *Fusobacterium* sp. 7_1 | 20 | 11 |  |
| GhaemL36 | NZ_ACDZ02000014 *Gemella haemolysans* ATCC 10379 | 29 | 24 |  |
| KoralL32 | NZ_ACJW02000003 *Kingella oralis* ATCC 51147 | 8 | 7 | NC_009655_2 |
| LbuccL29 | NC_013192 *Leptotrichia buccalis* DSM 1135 | 84 | 84 | NC_013192_2 |
| LbuccL37 | *Leptotrichia buccalis* DSM 1135 | 13 | 11 | NC_013192_8 |
| LcrisL29 | NC_014106 *Lactobacillus crispatus* ST1 | 16 | 13 | NC_014106_2 |
| LjassL36 | NZ_ACGO01000006 *Lactobacillus gasseri* JV-V03 | 20 | 19 |  |
| LjensL36 | NZ_GG704745 *Lactobacillus jensenii* 115-3-CHN | 34 | 32 |  |
| MhypeL30 | FP929048 *Megamonas hypermegale* ART12/1 | 11 | 10 |  |
| Neis_t014_L28 | NZ_GL349412 *Neisseria* sp. oral taxon 014 str. F0314 | 18 | 17 | NC_014125_1 |
| Neis_t014_L36 | NZ_GL349415 *Neisseria* sp. oral taxon 014 str. F0314 | 10 | 5 | NC_013016_1 |
| PacneL29 | NZ_ADFS01000004 *Propionibacterium acnes* J139 | 3 | 3 | NC_013530_9 |
| PbuccL36 | *Prevotella buccae* D17 | 10 | 10 |  |
| PcaroL28 | NZ_ABVY01000003 *Pectobacterium carotovorum* subsp. | 8 | 5 | NC_013421_4 |
| PmerdL32 | NZ_AAXE02000112 *Parabacteroides merdae* ATCC 43184 | 28 | 27 |  |
| PmicrL30 | NZ_ABEE02000017 *Peptostreptococcus micros* ATCC 33270 | 22 | 18 |  |
| PorisL30 | NZ_ACUZ02000034 *Prevotella oris* F0302 | 8 | 7 |  |
| PpropL29 | NC_008609 *Pelobacter propionicus* DSM 2379 | 44 | 40 | NC_002939_4 |
| Prev_t317_L37 | NZ_GG740072 *Prevotella* sp. oral taxon 317 str. F0108 | 89 | 63 |  |
| Prev_t472_L36 | NZ_ACZS01000076 *Prevotella* sp. oral taxon 472 | 18 | 16 |  |
| Prop_t191_L30 | NZ_ACVN01000017 *Propionibacterium* sp. oral taxon 191 | 20 | 19 |  |
| Prop_t191_L32 | NZ_ACVN01000017 *Propionibacterium* sp. oral taxon 191 | 74 | 73 |  |
| RdentL36 | NZ_ADVO01000018 *Rothia dentocariosa* ATCC 17931 | 17 | 15 | NC_014643_6 |
| RinteL36 | NZ_ABYJ01000523 *Roseburia intestinalis* L1-82 | 62 | 61 |  |
| RinteL36C2 | NZ_ABYJ01000388 *Roseburia intestinalis* L1-82 | 20 | 19 |  |
| RlactL32 | NZ_ABOU02000032 *Ruminococcus lactaris* ATCC 29176 | 25 | 25 |  |
| SgallL36 | NC_013798 *Streptococcus gallolyticus* UCN34 | 16 | 15 | NC_013798_1 |
| SmutaL36 | NC_013928 *Streptococcus mutans* NN2025 | 71 | 70 | NC_004350_1 |
| SnoxiL36 | NZ_ACKT01000005 *Selenomonas noxia* ATCC 43541 | 13 | 4 |  |
| SoralL35 | GG749268 *Streptococcus oralis* ATCC 35037 | 8 | 6 |  |
| Veil_sp3_1_44_L35 | NZ_GG770200 *Veillonella* sp. 3_1_44 | 14 | 11 | NC_013520_10 |
| Veil_sp3_1_44_L36 | NZ_GG770200 V*eillonella* sp. 3_1_44 | 9 | 6 | NC_013520_7 |
| Veil_sp6_1_27_L36 | NZ_GG770216 *Veillonella* sp. 6_1_27 | 11 | 9 |  |
| VvadeL39 | NZ_ABDE01000020 *Victivallis vadensis* ATCC BAA-548 | 14 | 14 |  |

Notes: For genomes that have multiple loci for a CRISPR (e.g., there are two FnuclL30 loci detected in *Fusobacterium nucleatum* subsp. polymorphum ATCC 10953), the largest locus was used for the statistics; some CRISPRs have small number of repeats because using contigs (instead of whole genome, which was not available; e.g., EyuriL30, Copr_spART55_L36, and Fuso_sp2_1_31_L36); Some CRISPRs tend to have very conserved repeats (e.g., *Leptotrichia buccalis* DSM 1135 has a CRISPR loci that has 84 identical copies of the LbuccL29 repeat).
