# Supplementary material for: Diverse CRISPRs Evolving in Human Microbiomes
Source: PLoS Genet. 2012 Jun 13;8(6):e1002441. doi: 10.1371/journal.pgen.1002441 (PMC3374615; doi:10.1371/journal.pgen.1002441)
Supplement: Table S4 — List of viral genomes and their accession IDs plotted in Figure 6A. (DOCX) [file pgen.1002441.s011.docx]

Table S4. List of viral genomes and their accession IDs plotted in Figure 6A.

| Accession ID | Genome name |
| --- | --- |
| NC_012756 | *Streptococcus* phage PH10 |
| NC_001825 | *Streptococcus* phage Cp-1 |
| NC_010945 | *Streptococcu*s phage PH15 |
| NC_004996 | *Streptococcus mitis* phage SM1 provirus |
| NC_005294 | Bacteriophage EJ-1 provirus |
| NC_003050 | *Streptococcus pneumoniae* bacteriophage MM1 provirus |
| NC_012753 | *Streptococcus* phage 5093 |
| NC_012884 | *Streptococcus* phage M102 |
| NC_002072 | *Streptococcus thermophilus* bacteriophage DT1 |
| NC_013645 | *Streptococcus phage Abc2* |
| NC_004584 | *Streptococcus* pyogenes phage 315.1 provirus |
| NC_004303 | *Streptococcus thermophilus* temperate bacteriophage O1205 |
| NC_000872 | *Streptococcus thermophilus* bacteriophage Sfi21 |
| NC_000871 | *Streptococcus thermophilus* bacteriophage Sfi19 |
| NC_002214 | *Streptococcus thermophilus* bacteriophage Sfi11 |
| NC_013598 | *Streptococcus* phage ALQ13.2 |
| NC_010353 | *Streptococcus* phage 858 |
| NC_007019 | *Streptococcus thermophilus* bacteriophage 2972 |
| NC_015274 | *Streptococcus phage Dp-1* |
| NC_009018 | *Siphoviridae Streptococcus phage phi3396* |
| NC_002072 | *Streptococcus thermophilus bacteriophage DT1* |
| NC_002185 | *Streptococcus thermophilus bacteriophage 7201* |
