# Supplementary material for: Diverse CRISPRs Evolving in Human Microbiomes
Source: PLoS Genet. 2012 Jun 13;8(6):e1002441. doi: 10.1371/journal.pgen.1002441 (PMC3374615; doi:10.1371/journal.pgen.1002441)
Supplement: Table S5 — List of viral genomes sharing high sequence similarities (≥90% identify over 30 bps) with CRISPR spacers. (DOCX) [file pgen.1002441.s012.docx]

Table S5. List of viral genomes sharing high sequence similarities (≥90% identify over 30 bps) with CRISPR spacers.

| CRISPR | Virus (identity or identity range) |
| --- | --- |
| LjensL36 | *Lactobacillus* phage Lv-1 (93–100%) |
| PacneL29 | *Propionibacterium* phage PA6 (94–100%) |
| GhaemL36 | *Streptococcus* phage Cp-1 (93%), *Streptococcus* phage PH10 (97%), *Streptococcus pneumoniae* bacteriophage MM1(97%) |
| Fuso_sp1_1_41FAA | *Cotesia congregata* virus segment (93%) |
| Fuso_sp2_1_31_L36 | *Staphylococcus* phage phiN315 provirus (91%) |
| Fuso_sp7_1_L30 | *Amsacta moorei* entomopoxvirus (93%), *Staphylococcus* phage phiPVL-CN125 (90%) |
| KoralL32 | A*ggregatibacter* phage S1249 (91–100%), Bacteriophage Aaphi23 (91–100%), Bacteriophage F108 (90–94%), Bacteriophage N15 virion (91%), Bacteriophage phi-MhaA1-PHL101 virus (91%), *Haemophilus* phage HP1 (90–100%), *Haemophilus* phage HP2 (90–100%), *Pseudomonas* phage 73 (90%), *Pseudomonas* phage phiKZ (90%) |
| Veil_sp3_1_44_L36 | Vernonia yellow vein betasatellite (93%) |
| CrectL30 | Spodoptera litura nucleopolyhedrovirus (94%) |
| LbuccL37 | Adoxophyes orana nucleopolyhedrovirus (94%), Bacteriophage Aeh1 (94%), *Clostridium botulinum* phage C-St (93%), Enterobacteria phage T4 (94%) |
| SoralL35 | *Streptococcus* phage Cp-1 (93%), *Streptococcus* phage PH10 (97–100%), *Streptococcus* pneumoniae bacteriophage MM1(100%) |
| FnuclL30 | Blueberry red ringspot virus (93%) |
| SRS018443L37 | Mycobacteriophage D29 (95%), *Mycobacterium* phage L5 (97%), *Mycobacterium* phage Omega virus (94%), *Rhizobium* phage 16-3 (91%) |
| SRS024132L35 | *Enterococcus* phage phiEf11 (90%), *Pseudomonas* phage phiKZ (93%) |
| SRS018394L37 | Acanthamoeba polyphaga mimivirus (90%), Bacteriophage G1 (90%), Cyanophage P-SSM2 (93%), Hemorrhagic enteritis virus 94%), Human papillomavirus type 48 (90%), *Staphylococcus* phage K virion (90%), Turkey adenovirus 3 (94%) |
| SRS011126L30 | Bacteriophage phi1026b (91%), Bacteriophage phiE125 (91%), *Burkholderia* phage phi644-2 (91%) |
| SRS015378L34 | *Actinomyces* phage Av-1 virus (94%) |
| SRS064449L29 | *Staphylococcus* phage Twort (94%) |
| SRS019591L36 | *Lactobacillus* phage Lb338-1 (93%), *Lactobacillus plantarum* bacteriophage LP65 (93%), *Listeria* phage A511 (93%), *Staphylococcus* phage Twort (93%) |
| SRS062761L37 | Alcelaphine herpesvirus 1 (90%), Ateline herpesvirus 3 (91-94%), Gallid herpesvirus 2 (90%), Koi herpesvirus (90%), Murid herpesvirus 2 (91%), Ralstonia phage RSL1 (93%), Shrimp white spot syndrome (90%) |
| SRS012279L36 | *Acanthamoeba polyphaga* mimivirus (90%) |
| SRS014470L37 | *Lactobacillus plantarum* bacteriophage LP65 (97%) |
