# Supplementary material for: Diverse CRISPRs Evolving in Human Microbiomes
Source: PLoS Genet. 2012 Jun 13;8(6):e1002441. doi: 10.1371/journal.pgen.1002441 (PMC3374615; doi:10.1371/journal.pgen.1002441)
Supplement: Table S6 — List of plasmids sharing high sequence similarities (≥90%) with CRISPR spacers. (DOCX) [file pgen.1002441.s013.docx]

Table S6. List of plasmids sharing high sequence similarities (≥90%) with CRISPR spacers.

| CRISPR ID | Plasmids |
| --- | --- |
| LjassL36 | *Lactobacillus casei* str. Zhang plasmid plca36 |
| FperiL30 | *Fusobacterium nucleatum* plasmid pFN1, *Fusobacterium nucleatum* plasmid pPA52, *Fusobacterium nucleatum* subsp. polymorphum ATCC 10953 plasmid pFN3 |
|  |  |
|  |  |
| Fuso_sp1_1_41FAA_L36 | *Fusobacterium nucleatum* plasmid pFN1, *Fusobacterium nucleatum* plasmid pKH9, *Fusobacterium nucleatum* plasmid pPA52, *Fusobacterium nucleatum* subsp. polymorphum ATCC 10953 plasmid pFN3 |
| Fuso_sp7_1_L30 | *Clostridium perfringens* plasmid pCPF4969, *Enterococcus faecalis* plasmid pAMbeta1, *Enterococcus faecalis* plasmid pRE25, *Enterococcus faecalis* plasmid pWZ1668, *Lactococcus garvieae* plasmid pKL0018, *Staphylococcus aureus* plasmid pV030-8 |
| KoralL32 | *Actinobacillus actinomycetemcomitans* plasmid pVT745, *Actinobacillus pleuropneumoniae* plasmid p9555, *Actinobacillus pleuropneumoniae* plasmid p9956, *Actinobacillus pleuropneumoniae* plasmid pARD3079, *Actinobacillus pleuropneumoniae* plasmid pKMA2425, *Actinobacillus pleuropneumoniae* plasmid pTYM1, *Actinobacillus porcitonsillarum* plasmid pKMA757, *Avibacterium paragallinarum* plasmid pYMH5, *Eikenella corrodens* plasmid pMU1*, Haemophilus ducreyi* plasmid pNAD1, *Haemophilus influenzae* biotype aegyptius plasmid pF3031, *Haemophilus influenzae* plasmid ICEhin1056, *Haemophilus parasuis* plasmid pHS-Tet, *Mannheimia haemolytica* plasmid pCCK3259, *Mannheimia haemolytica* plasmid pMHSCS1, *Pasteurella multocida* plasmid pB1005, *Pasteurella multocida* plasmid pB1006, *Pasteurella multocida* plasmid pCCK647, *Pasteurella multocida* plasmid pIG1, *Pseudomonas putida* plasmid pWW0, *Pseudomonas* sp. ND6 plasmid |
| Veil_sp3_1_44_L36 | *Escherichia coli* plasmid pO113, *Staphylococcus chromogenes* plasmid pLNU1,  *Sulfolobus islandicus* plasmid pXZ1 |
| LbuccL37 | *Bacillus megaterium* plasmid pBM400 |
| FalocL36 | *Campylobacter coli* plasmid pCC31, *Campylobacter jejuni* plasmid pTet, *Pasteurella multocida* plasmid pB1006 |
| RinteL36 | *Campylobacter coli* plasmid pCC31, *Campylobacter jejuni* plasmid pTet, Uncultured bacterium plasmid pTRACA20 |
| CmatrL29 | *Corynebacterium casei* plasmid pCASE1, *Corynebacterium glutamicum* plasmid pXZ10145.1, *Corynebacterium glutamicum* strain 1014 plasmid pXZ10142 |
| SoralL35 | *Bacillus megaterium* plasmid pBM400, *Enterococcus faecalis* plasmid pCF10 |
| EamylL29 | *Escherichia coli* plasmid plLF82 |
| LbuccL29 | *Bacillus thuringiensis* plasmid pBMB67 |
| Neis_t014_L28 | *Eikenella corrodens* plasmid pMU1 |
| SmutaL36 | *Bacillus pumilus* plasmid pPL10, *Bacillus subtilis* plasmid pTA1040, *Lactococcus lactis* plasmid pSK11B, *Streptococcus parasanguinis* plasmid pFW213, *Streptococcus pneumoniae* plasmid pDP1, *Streptococcus pneumoniae* plasmid pSMB1, *Streptococcus pneumoniae* plasmid pSpnP1, *Streptococcus thermophilus* plasmid pER35, *Streptococcus thermophilus* plasmid pER36, *Streptococcus thermophilus* plasmid pND103, *Streptococcus thermophilus* plasmid pSMQ173b, *Streptococcus thermophilus* plasmid pSMQ308 |
| Neis_t014_L36 | *Burkholderia cepacia* plasmid pIJB1, *Escherichia coli* plasmid pAR060302, *Escherichia coli* plasmid peH4H, *Neisseria gonorrhoeae* plasmid pCmGFP, N*eisseria gonorrhoeae* plasmid pEP5289, *Neisseria gonorrhoeae* plasmid pJD1, *Neisseria lactamica plasmid* pNL01, *Neisseria lactamica* plasmid pNL3.2, *Neisseria lactamica* plasmid pNL9, *Neisseria meningitidis* plasmid pJS-B, *Pseudomonas aeruginosa* plasmid pMATVIM-7, *Salmonella enterica* plasmid pAM04528, *Shigella flexneri* virulence plasmid, Uncultured bacterium plasmid pB10 |
| SRS018394L37 | *Bacillus megaterium* plasmid pBM400, *Borrelia burgdorferi* 297 plasmid, *Borrelia burgdorferi* JD1 plasmid, *Borrelia burgdorferi* N40 plasmid, *Fusobacterium nucleatum* plasmid pFN1, *Fusobacterium nucleatum* plasmid pPA52 |
| SRS018394L36 | *Neisseria gonorrhoeae* plasmid pEP5289 |
| SRS011126L30 | *Campylobacter lari* plasmid pCL300 |
| SRS020226L38 | *Haemophilus influenzae* plasmid ICEhin1056 |
| SRS018975L36 | *Rhodococcus erythropolis* plasmid pBD2 |
| SRS019591L36 | *Bacillus* sp. BS-01 plasmid, *Enterococcus faecalis* plasmid pAMbeta1, *Enterococcus faecalis* plasmid pRE25, *Enterococcus faecalis* plasmid pWZ1668, *Enterococcus faecium* plasmid pRUM, *Lactobacillus plantarum* plasmid pLFE1, *Lactobacillus reuteri* plasmid pTE44, *Lactobacillus reuteri* strain AE78 plasmid pAE78, *Lactococcus garvieae* plasmid pKL0018, *Pediococcus acidilactici* plasmid pEOC01, *Streptococcus agalactiae* plasmid pGB3631, *Streptococcus pyogenes* plasmid pSM19035 |
| SRS062761L37 | *Pyrococcus* sp. 12/1 plasmid |
| SRS048791L36 | *Bacillus megaterium* plasmid pBM400 |
| SRS042131L36 | *Bacillus megaterium* plasmid pBM400 |
| SRS013506L37 | *Actinobacillus actinomycetemcomitans* plasmid pVT745,  *Haemophilus influenzae* biotype aegyptius plasmid pF3031  *Haemophilus influenzae* plasmid ICEhin1056 |
| SRS057478L36 | Uncultured bacterium plasmid pTRACA17 |
