# Supplementary material for: Diverse CRISPRs Evolving in Human Microbiomes
Source: PLoS Genet. 2012 Jun 13;8(6):e1002441. doi: 10.1371/journal.pgen.1002441 (PMC3374615; doi:10.1371/journal.pgen.1002441)
Supplement: Table S7 — Targeted assembly results of 10 CRISPRs using reads simulated from 6 genomes. (DOCX) [file pgen.1002441.s014.docx]

Table S7. Targeted assembly results of 10 CRISPRs using reads simulated from 6 genomes.

| Genome name | CRISPR repeat | CRISPR coordinates | Contig coordinates |
| --- | --- | --- | --- |
| *Azospirillum B510* (NC_013854) | GTTGCGGCTGGACCCCCGATCCCCATCGGCTACACT | 1016560–1018180 | 1016567–1016758  1016783–1017407  1017652–1018045 |
|  | GCTTCAATGAGGCCCAAGCATTTCTGCCTGGGAAGAC | 3034463–3034649  3035919–3038413 | 3035963–3036203  3036310–3036729  3036936–3037225  3037421–3037629  3037766–3037964  3038122–3038299 |
|  | CCCTTCCTGGGCGGAAACGCCCAGGCCTCATTGAAGC | 3158606–3163849  3199247–3200867 | 3158730–3158933  3159098–3159532  3159568–3159810  3160022–3160322  3161040–3161536  3161580–3161917  3161947–3162899  3163117-3163322  3199499–3199738  3200064–3200530  3200623–3200763 |
| *Streptococcus mutans* NN2025 (NC_013928) | ATTTTACCCGCACGAGCGGGGGTGATCCT | 644279–645406 | 644186–645437 |
|  | GTTTTAGAGCTGTGTTGTTTCGAATGGTTCCAAAAC | 743991–748582 | 743900–746138  746189–747600  747556–747657  747613–747732  747745–748459  748415–748525 |
| *Deferribacter desulfuricans* SSM1 (NC_013939) | GTTTCAATTCCTCATAGGCACTCTAAAAAC | 514731–516696  526129–527564 | 514710–516792  526104–526507  526580–527653 |
| *Dehalococcoides* GT (NC_013890) | CGGTTCACCCCCACATGCGTGGGGAATAC | 1176477–1178766 | 1176384–1178812 |
| *Erwinia amylovora* ATCC 49946 (NC_013971) | GTGTTCCCCGCGTGAGCGGGGATAAACCG | 854678–856841  867748–869731 | 854580–854799  854874–855088  855150–856812  867671–868424  868419–868817  868889–869702 |
|  | GTTCACTGCCGTACAGGCAGCTTAGAAA | 879195–879523 | 879124–879293  879330–879468 |
| *Escherichia coli K12* MG1655 (NC_000913) | GGTTTATCCCCGCTGGCGCGGGGAACAC | 2875723–2876485 | 2875708–2876577 |
